# Safety of Simultaneous Vaccination With Adjuvanted Zoster Vaccine and Adjuvanted Influenza Vaccine: A Randomized Clinical Trial

**DOI:** 10.1001/jamanetworkopen.2024.40817

**Published:** 2024-10-24

**Authors:** Kenneth E. Schmader, Emmanuel B. Walter, Kawsar R. Talaat, Wes Rountree, Marek Poniewierski, Emily Randolph, Sean X. Leng, Bettina Wunderlich, Michael M. McNeil, Oidda Museru, Karen R. Broder

**Affiliations:** 1 Division of Geriatrics, Department of Medicine and Center for the Study of Aging, Duke University School of Medicine, Durham, North Carolina; 2 Geriatric Research Education and Clinical Center, Durham Veterans Affairs Health Care System, Durham, North Carolina; 3 Duke Human Vaccine Institute, Duke University School of Medicine, Durham, North Carolina; 4 Department of Pediatrics, Duke University School of Medicine, Durham, North Carolina; 5 Department of International Health, Johns Hopkins University Bloomberg School of Public Health, Baltimore, Maryland; 6 Division of Geriatric Medicine and Gerontology, Department of Medicine, Johns Hopkins University School of Medicine, Baltimore, Maryland; 7 Johns Hopkins Center on Aging and Immune Remodeling, Johns Hopkins University School of Medicine and Bloomberg School of Public Health, Baltimore, Maryland; 8 Immunization Safety Office, Centers for Disease Control and Prevention, Atlanta, Georgia; 9 Influenza Division, Centers for Disease Control and Prevention, Atlanta, Georgia

## Abstract

**Importance:**

Quadrivalent adjuvanted inactivated influenza vaccine (aIIV4) and adjuvanted recombinant zoster vaccine (RZV) contain novel adjuvants. Data are limited on the comparative safety, reactogenicity, and health-related quality of life (HRQOL) effects of the simultaneous administration of these vaccines.

**Objective:**

To compare the safety and reactogenicity after simultaneous doses of RZV and aIIV4 administration (opposite arms) with simultaneous doses of RZV with quadrivalent high-dose inactivated influenza vaccine [HD-IIV4]).

**Design, Setting, and Participants:**

This randomized blinded clinical trial was conducted during the 2021-2022 and 2022-2023 influenza seasons at 2 centers in the US among community-dwelling adults aged 65 years or older. Analysis was performed on an intention-to-treat basis.

**Intervention:**

Simultaneous intramuscular administration of RZV dose 1 and aIIV4 or HD-IIV4 in opposite arms after age stratification (65-69 and ≥70 years) and randomization.

**Main Outcomes and Measures:**

The primary outcome was the proportions of participants with 1 or more severe solicited reactions during days 1 to 8, using a noninferiority test (10% noninferiority margin). Additional measures included serious adverse events and adverse events of clinical interest during days 1 to 43 of the study period.

**Results:**

A total of 267 adults (median age, 71 years [range, 65-92 years]; 137 men [51.3%]) were randomized; 130 received simultaneous RZV and aIIV4, and 137 received simultaneous RZV and HD-IIV4. The proportion of patients reporting 1 or more severe reactions after simultaneous administration of RZV and aIIV4 (15 of 115 [11.5%]) was noninferior compared with simultaneous RZV and HD-IIV4 (17 of 119 [12.5%]) (absolute difference, −1.0% [95% CI, −8.9% to 7.1%]). There were no significant differences in the number of serious adverse events or adverse events of clinical interest between the groups.

**Conclusions and Relevance:**

In this clinical trial of simultaneous doses of RZV and aIIV4 compared with simultaneous doses of RZV and HD-IIV4, overall safety findings were similar between groups. From a safety standpoint, this study supports the simultaneous administration of RZV and aIIV4 among older adults.

**Trial Registration:**

ClinicalTrials.gov Identifier: NCT05007041

## Key Points

**Question:**

What is the safety of the simultaneous administration of 2 vaccines containing novel adjuvants, recombinant zoster vaccine (RZV) and quadrivalent adjuvanted inactivated influenza vaccine (aIIV4), among adults aged 65 years or older?

**Findings:**

In this randomized clinical trial comprising 267 older adults, the proportion of participants with at least 1 severe solicited reactogenicity event was noninferior in the simultaneous RZV and aIIV4 group compared with the simultaneous RZV and quadrivalent high-dose inactivated influenza group.

**Meaning:**

This study suggests that, from a safety standpoint, the simultaneous administration of RZV and aIIV4 is an acceptable option for vaccine delivery among older adults.

## Teaser

This randomized clinical trial compares the safety and reactogenicity after simultaneous doses of adjuvanted recombinant zoster vaccine and adjuvanted inactivated influenza vaccine vs simultaneous doses of adjuvanted recombinant zoster vaccine and high-dose inactivated influenza vaccine.

## Introduction

Novel nonaluminum adjuvants are powerful immunostimulants used in vaccine platforms to improve immunogenicity and efficacy.^1^ In recent years, the US Food and Drug Administration (FDA) licensed several vaccines with novel adjuvants.^2,3,4,5,6^ Clinicians may opt to administer these vaccines simultaneously when indicated. Vaccines with novel adjuvants are more reactogenic than vaccines without adjuvants, and there is a theoretical possibility that novel adjuvants could activate immune-mediated disease in some individuals.^7^ Data are limited on the safety of the simultaneous administration of vaccines with novel adjuvants.

For older adults, the need for safety data on simultaneous administration of vaccines with novel adjuvants has specific clinical relevance. An adjuvanted recombinant zoster vaccine (RZV) containing the novel adjuvant AS01B, monophosphoryl lipid A and saponin, and recombinant glycoprotein E was licensed in 2017 and recommended by the Centers for Disease Control and Prevention (CDC) Advisory Committee on Immunization Practices (ACIP) for the prevention of herpes zoster in immunocompetent adults aged 50 years or older.^5,8^ A trivalent adjuvanted influenza vaccine containing the novel adjuvant MF-59, a squalene-based oil-in-water emulsion, was licensed in 2015 for the prevention of influenza in adults aged 65 years or older,^9^ and in 2020, the adjuvanted quadrivalent formulation (aIIV4) was licensed.^3^ Since 2022, the ACIP has recommended that adults aged 65 years or older preferentially receive any one of the following: quadrivalent high-dose inactivated influenza vaccine (HD-IIV4),^10^ quadrivalent recombinant influenza vaccine,^11^ or aIIV4.^3,12^ The safety of simultaneous administration of aIIV and RZV has not been assessed in a clinical trial, to our knowledge.

We performed a randomized clinical trial to determine the safety of simultaneous doses of RZV and aIIV4 compared with simultaneous doses of RZV and HD-IIV4 among adults aged 65 years or older. The primary objective was to compare the proportion of participants with at least 1 severe (grade 3) solicited local or systemic reactogenicity event after RZV dose 1 in the RZV and aIIV4 group vs the RZV and HD-IIV4 group.

## Methods

### Study Design and Participants

We conducted a prospective, randomized, blinded clinical trial at CDC-sponsored Clinical Immunization Safety Assessment (CISA) Project sites^13^ at Duke University and Johns Hopkins Bloomberg School of Public Health during the 2021-2022 and 2022-2023 influenza seasons. The study protocol was approved by institutional review boards at each study site and was registered at ClinicalTrials.gov (NCT05007041); the CDC did not use its own institutional review board, but instead approved the use of the Duke University institutional review board. The trial protocol and statistical analysis plan are provided in Supplement 1. Eligibility criteria included being 65 years or older, living in the community, having no immunosuppression, being without dementia, being able to speak English, and having no contraindications to influenza or RZV vaccination (eMethods 1 in Supplement 2). The trial followed the Consolidated Standards of Reporting Trials (CONSORT) reporting guideline for parallel group randomized clinical trials. Participants provided written informed consent on day 1.

Study staff screened potential participants for cognitive impairment with the Mini-Cog.^14^ Staff members collected data on demographics, self-identified race and ethnicity (assessed to provide information on the generalizability of the findings to the patient population), medical history, medication, and influenza and zoster immunization for each participant. Participants were randomized (1:1) to receive simultaneous RZV dose 1 and aIIV4 or simultaneous RZV dose 1 and HD-IIV4 using a permuted block randomization scheme stratified by study site (day 1). Separate permuted block randomization schemes were used for participants aged 65 to 69 years and those aged 70 years or older. Participants and study staff performing data collection and analysis were blinded to treatment allocation for influenza vaccine using a permuted block randomization scheme that was uploaded to the Research Electronic Data Capture (REDCap) platform; RZV was administered open label. Because there was a visual difference between aIIV4 and HD-IIV4, staff members who prepared and administered study vaccines were unblinded but did not participate in data collection, outcome measurement, or analysis.

After randomization, a 0.5-mL intramuscular dose of RZV (dose 1) and a 0.5-mL intramuscular dose of aIIV4 were administered in the deltoid muscle in opposite arms or a 0.5-mL intramuscular dose of RZV (dose 1) and a 0.7-mL intramuscular dose of HD-IIV4 were administered in the deltoid muscle in opposite arms. Each aIIV4 dose contained 15 μg of hemagglutinin from each of the 4 recommended influenza strains for the respective season and MF59 adjuvant. Each HD-IIV4 dose contained 60 μg of hemagglutinin from each of the 4 recommended influenza strains for the respective season. We followed up with participants during the study through approximately 3 months after vaccination. Participants received RZV dose 2 without other vaccines on day 60.

### Safety and Reactogenicity Assessments

Study staff members monitored participants in the clinic for 15 minutes or longer after vaccination for immediate adverse events, including anaphylaxis and syncope, and assessed solicited reactogenicity events and unsolicited adverse events after vaccination on day 1 (vaccination day) through day 8 using a standard symptom paper diary. The local reactions assessed included injection-site pain, swelling, and redness in both arms. The systemic reactions assessed included fever, chills, fatigue, myalgia, arthralgia, headache, and gastrointestinal symptoms (nausea, vomiting, diarrhea, and/or abdominal pain). Participants received a study thermometer, ruler, and education about completing the diary. Participants graded the severity of their reactions based on criteria in prelicensure trials of aIIV4, HD-IIV4, and RZV as none (grade 0), mild (grade 1), moderate (grade 2), or severe (grade 3)^3,5,10^ (eTable 1 in Supplement 2). Study staff members contacted participants on day 3 and day 9 after vaccination to review solicited reactogenicity data and assess for unsolicited adverse events, serious adverse events (SAEs), and adverse events of clinical interest (AECIs), as well as any new medical conditions or change in medications. Staff members also monitored study participants for these outcomes, except for solicited reactogenicity, through day 43 and throughout the study period. Serious adverse events were defined in accordance with the FDA.^15^ Adverse events of clinical interest included syncope during postvaccination monitoring in clinic, anaphylaxis within 24 hours after vaccination, and new-onset, immune-mediated conditions (including Guillain-Barré syndrome [GBS]) during the study. Study investigators assessed relatedness of SAEs, AECIs, and adverse events to the study vaccines, based on their judgment, with consultation from experts on a study safety panel as needed.

### Health-Related Quality-of-Life Assessments

Health-related quality of life (HRQOL) was assessed after vaccination on day 1 (in clinic) and daily for 7 days using the EuroQol 5 Dimensions-5 Level (EQ-5D-5L)^16^ and EuroQol Visual Analogue Scale (EQ-VAS)^17^ (eMethods 2 in Supplement 2). The EQ-5D-5L is a standardized, generic measure of health status.^16^ The EQ-VAS measures the respondent’s self-rated health status (higher scores are better).^17^ Permission was obtained to use the EuroQol measures.

### Outcome Measures

The primary outcome was a comparison of the proportions of participants with at least 1 severe (grade 3) solicited reactogenicity event on days 1 to 8 after RZV dose 1 in each study group. We hypothesized that the proportion of participants with at least 1 severe (grade 3) solicited reactogenicity event would be noninferior (not higher) in the simultaneous RZV dose 1 and aIIV4 group compared with the simultaneous RZV dose 1 and HD-IIV4 group. Secondary outcomes included a comparison of the proportion of participants with at least 1 severe (grade 3) solicited local or systemic reactogenicity event in the 2 groups and the proportion of participants with at least 1 SAE or AECI in the 2 groups through day 43.

Exploratory outcomes included the proportion of participants with at least 1 moderate to severe (grades 2 and 3) solicited local or systemic reactogenicity event on days 1 to 8 after RZV dose 1, the proportion of participants with SAEs and AECIs after RZV dose 2, and clinical description of these events in each study group through the entire study period and change in scores on the HRQOL assessments before and after vaccination (for visit 1) in each group.

### Statistical Analysis

The planned sample size of at least 380 evaluable participants (190 in each group across the study sites) provided at least 80% power to reject the null hypothesis that the proportion of participants with at least 1 severe (grade 3) solicited reactogenicity event would be inferior in the RZV and aIIV4 group compared with the RZV and HD-IIV4 group. The statistical testing for the primary outcome was conducted at the 1-sided α level of .03 using the upper bound of a stratified-by-site Newcombe binomial confidence interval^18^ with Cochran-Mantel-Haenszel weighting with a noninferiority margin of 10%. This statistical method was also used for the comparisons of the difference in at least 1 severe (grade 3) solicited local or systemic reactogenicity event separately and for comparison of the proportions of moderate to severe reactogenicity events on days 1 to 8 after RZV dose 1 in each study group. As stated in the protocol, the reactogenicity comparisons were made using a modified intention-to-treat (mITT) population, defined as all participants who were randomized, were vaccinated, and provided at least 1 day of complete data on the symptom diary form; the other comparisons were made using an intention-to-treat (ITT) population, including all participants who were randomized and vaccinated.

The comparison of the frequencies of SAEs and AECIs in the 2 treatment groups was made using exact binomial 95% CIs and a 95% CI of the difference using the ITT population, defined as all participants who were randomized and vaccinated. Linear regression modeling was used to compare changes from baseline for HRQOL outcomes, and Mann-Whitney tests were used to compare the maximum difference score from baseline. These data were analyzed using SAS/STAT software, version 9.4 (SAS Institute Inc).

## Results

### Study Participants

**Figure 1.**
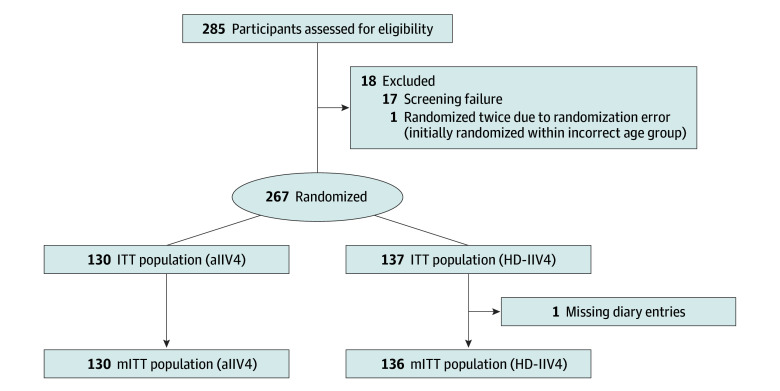
CONSORT Diagram Randomization and patient flow in the study comparing recombinant zoster vaccine dose 1 and quadrivalent adjuvanted inactivated influenza vaccine (aIIV4) vs recombinant zoster vaccine dose 1 and quadrivalent high-dose inactivated influenza vaccine (HD-IIV4). The intention-to-treat (ITT) population consisted of all participants who were randomized and vaccinated. The modified ITT (mITT) population consisted of all participants who were randomized, vaccinated, and provided at least 1 day of complete data on the symptom diary form.

A total of 267 adults (median age, 71 years [range, 65-92 years]; 137 men [51.3%] and 130 women [48.7%]; 1 Asian participant [0.4%], 19 Black participants [7.1%], 3 Hispanic participants [1.1%], and 247 White participants [92.5%]) were randomized; 130 received simultaneous RZV dose 1 and aIIV4, and 137 received simultaneous RZV dose 1 and HD-IIV4 in the ITT population (Figure 1). We randomized 102 participants in 2021-2022 and 165 participants in 2022-2023. For the mITT population, 130 received simultaneous RZV dose 1 and aIIV4, and 136 received simultaneous RZV dose 1 and HD-IIV4; 1 participant lacked symptom diary data (Figure 1). Baseline demographic and clinical characteristics were similar between the 2 study groups (Table 1).

**Table 1.** Baseline Demographic and Clinical Characteristics of Participants

Characteristic	Participants, No. (%)	
	RZV dose 1 and aIIV4 (n = 130)	RZV dose 1 and HD-IIV4 (n = 137)
Study site		
Duke University	108 (83.1)	113 (82.5)
Johns Hopkins University	22 (16.9)	24 (17.5)
Influenza season of enrollment		
2021-2022	49 (37.7)	53 (38.7)
2022-2023	81 (62.3)	84 (61.3)
Age, median (range), y	71.5 (65-92)	71.0 (65-87)
65-69	44 (33.8)	48 (35.0)
≥70	86 (66.2)	89 (65.0)
Sex		
Female	61 (46.9)	69 (50.4)
Male	69 (53.1)	68 (49.6)
Race		
Asian	0	1 (0.7)
Black	7 (5.4)	12 (8.8)
White	123 (94.6)	124 (90.5)
Other^a^	0	0
Ethnicity		
Hispanic	2 (1.5)	1 (0.7)
Cardiovascular and respiratory disorders^b^		
Atrial fibrillation	7 (5.4)	5 (3.6)
Coronary artery disease	5 (3.8)	12 (8.8)
Heart failure	0	1 (0.7)
Hyperlipidemia	17 (13.1)	26 (19.0)
Hypertension	33 (25.4)	51 (37.2)
Valvular heart disease	2 (1.5)	3 (2.2)
Asthma	6 (4.6)	7 (5.1)
Chronic obstructive pulmonary disease	2 (1.5)	2 (1.5)
Other common conditions		
Arthritis	16 (12.3)	25 (18.2)
Depression	10 (7.7)	8 (5.8)
Diabetes	14 (10.8)	18 (13.1)
Gastroesophageal reflux disease	9 (6.9)	7 (5.1)
Hearing loss	8 (6.2)	7 (5.1)
Hypothyroidism	13 (10.0)	11 (8.0)
Prevaccination EQ-5D-5L score, mean (SD)^c^	87.8 (9.6)	87.2 (9.8)
Prevaccination EQ-VAS score, mean (SD)^d^	0.92 (0.11)	0.89 (0.11)

Abbreviations: aIIV4, adjuvanted inactivated influenza vaccine, quadrivalent; EQ-5D-5L, EuroQol 5 Dimensions-5 Level; EQ-VAS, EuroQol Visual Analogue Scale; HD-IIV4, high-dose inactivated influenza vaccine, quadrivalent; RZV, recombinant zoster vaccine.

^a^ Includes American Indian or Alaskan Native, and more than 1 race.

^b^ These conditions are not mutually exclusive.

^c^ The score is a utility index that ranges from −0.109 (worst health) to 1.000 (best health) for US-specific values.

^d^ EQ-VAS measures self-rated health with a score that ranges from 0 to 100, with 100 equal to the “best health you can imagine” and 0 equal “to the worst health you can imagine.”

### Safety and Reactogenicity

**Figure 2.**
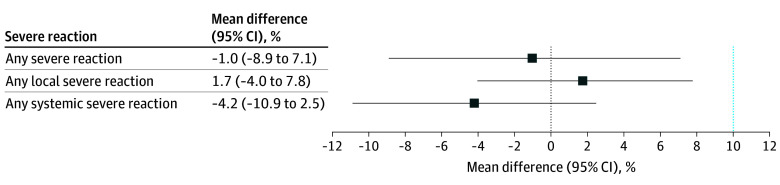
Difference in Proportions for Any Severe, Local, or Systemic Reactions From Recombinant Zoster Vaccine (RZV) Dose 1 and Quadrivalent Adjuvanted Inactivated Influenza Vaccine (aIIV4) vs RZV Dose 1 and Quadrivalent High-Dose Inactivated Influenza Vaccine (HD-IIV4) Mean differences and 95% CIs in proportions of severe (grade 3) reactions (any, local, or systemic) after RZV dose 1 and aIIV4 minus RZV dose 1 and HD-IIV4 are shown. The vertical dotted line at 0 indicates no difference, and the vertical dotted line at 10 indicates the 10% noninferiority margin. Error bars indicate 95% CIs.

The number and proportion of participants experiencing local or systemic reactions (mild, moderate, or severe) after simultaneous vaccination with RZV dose 1 and aIIV4 or HD-IIV4 are shown in in eTable 2 in Supplement 2. The proportion of participants reporting at least 1 severe (grade 3) solicited reactogenicity event was noninferior (not higher) in the RZV and aIIV4 group (15 of 115 [11.5%]) compared with the simultaneous RZV and HD-IIV4 group (17 of 119 [12.5%]) (absolute difference, −1.0% [95% CI, −8.9% to 7.1%]) (Figure 2). The proportion of participants reporting at least 1 severe (grade 3) solicited local reactogenicity event was noninferior in the RZV and aIIV4 group (8 of 122 [6.2%]) compared with the RZV and HD-IIV4 group (6 of 130 [4.4%]) (absolute difference, 1.7% [95% CI, −4.0% to 7.8%]). The proportion of participants reporting at least 1 severe (grade 3) solicited systemic reactogenicity event was noninferior in the RZV and aIIV4 group (7 of 123 [5.4%]) compared with the simultaneous RZV and HD-IIV4 group (13 of 123 [9.6%]) (absolute difference, −4.2% [95% CI, −10.9% to 2.5%]) (Figure 2). The proportion of participants reporting at least 1 severe (grade 3) solicited reactogenicity event, local reactogenicity event, or systemic reactogenicity event was also noninferior after RZV dose 2 (administered without other vaccines) in the 2 groups (eTable 3 in Supplement 2). The frequency of moderate to severe reactions was similar when RZV dose 1 was administered at the same visit with aIIV4 or HD-IIV4 (eTable 2 in Supplement 2). Recombinant zoster vaccine dose 1 or dose 2 and aIIV4 did not meet the noninferiority criteria compared with RZV dose 1 or dose 2 and HD-IIV4 for any moderate to severe reaction (eTable 4 in Supplement 2). No participant sought medical attention for a local or systemic reaction after vaccination on days 1 to 8 after RZV dose 1 or dose 2. We observed clinically similar patterns of severe reactions among participants aged 65 to 69 years and those aged 70 years or older (eTable 5 in Supplement 2).

There were no episodes of syncope during postvaccination monitoring in the clinic or anaphylaxis within 24 hours of vaccination. During the 43-day follow-up period, there were no episodes of GBS or deaths. During the 43 days after visit 1, 1 of 130 participants in the RZV and aIIV4 group had an SAE (0.8% [95% CI, 0.02%-4.2%]); 5 of 137 participants in the RZV and HD-IIV4 group had an SAE (3.7% [95% CI, 1.2%-8.3%]) (Table 2). The difference between the aIIV4 and HD-IIV4 groups was −2.9 (95% CI, −6.4 to 0.6). Study investigators, with input from the safety panel, assessed 1 SAE, a left partial cranial nerve III palsy, as possibly related to vaccination in the RZV and HD-IIV4 group as a new-onset, immune-mediated condition and, therefore, an AECI. Throughout the entire study period, there were 9 participants reporting SAEs: 4 of 130 in the RZV and aIIV4 group (3.1%) and 5 of 137 in the RZV and HD-IIV4 group (3.7%). The difference between the aIIV4 and HD-IIV4 groups was −0.6 (95% CI, −4.9 to 3.8). Three of the participants had onset of the SAE after day 43 (Table 2). Two of these participants received RZV dose 2, and the other did not. There were no cases of GBS or deaths during the study period after RZV dose 2.

**Table 2.** Older Adults Experiencing Serious Adverse Events Throughout the Study

RZV dose 1 and influenza vaccine group	Age group, y	Time since vaccination with dose 1 RZV and influenza vaccine, d^a^	Relatedness	Serious adverse event description
HD-IIV4^b^	65-69	26	Not related	Cerebrovascular accident
HD-IIV4^b^	≥70	21	Not related	Acute hyperkalemia
HD-IIV4^b^	≥70	25	Not related	Shortness of breath
HD-IIV4^b^	≥70	37	Not related	Pulmonary embolism
HD-IIV4	65-69	25	Possibly related	Left partial cranial nerve III palsy^c^
aIIV4^b^	≥70	41	Not related	Arrhythmia
aIIV4^d^	≥70	73	Not related	Hepatocellular carcinoma
aIIV4^b^	≥70	98	Not related	Revision of right shoulder rotator cuff surgery
aIIV4^b^	65-69	57	Not related	Chronic obstructive pulmonary disease exacerbation

Abbreviations: aIIV4, adjuvanted inactivated influenza vaccine, quadrivalent; HD-IIV4, high-dose inactivated influenza vaccine, quadrivalent; RZV, recombinant zoster vaccine.

^a^ Vaccination day is day 1 (eg, 26 days after vaccination means the event occurred on day 27).

^b^ Participant received RZV dose 2 at approximately day 60.

^c^ This serious adverse event was judged to be possibly related to receipt of vaccine; the palsy resolved completely by 8 weeks after the hospitalization (1-day duration); the participant did not receive RZV dose 2 during the study.

^d^ Participant did not receive RZV dose 2 in the study.

### Health-Related Quality of Life

Participants’ baseline prevaccination (visit 1) HRQOL scores were similar in both study groups (Table 1). Among all participants, there were minimal changes in the EQ-5D-5L Utility Index Score (eFigure 1 in Supplement 2) and EQ-VAS score (eFigure 2 in Supplement 2) from day 1 through day 8 after vaccination; changes were similar between treatment groups. Among participants with severe (grade 3) reactions, there was a decrease in the EQ-5D-5L Utility Index Score and EQ-VAS scores after day 1 that reached a nadir at day 2 and recovered to baseline by days 3 and 4 (eFigure 2 in Supplement 2). No group differences were detected using linear regression for the maximum decrease from baseline. For the EQ-5D-5L Utility Index, the maximum decrease was −0.08 for aIIV4 vs −0.07 for HD-IIV4 (difference, −0.01 [95% CI, −0.03 to 0.01]; *P* = .31). For the EQ-VAS score, the maximum decrease was −7.64 for aIIV4 vs −7.72 for HD-IIV4 (difference, −0.08 [95% CI, −2.62 to 2.79]; *P* = .82).

## Discussion

To our knowledge, this is the first randomized clinical trial in the US directly comparing the safety and reactogenicity after the simultaneous administration of 2 vaccines containing novel adjuvants, RZV and aIIV4, among adults aged 65 years or older. The CDC ACIP recommends that “…selection of a nonadjuvanted influenza vaccine may be considered in situations in which influenza vaccine and another vaccine containing a nonaluminum adjuvant are to be administered concomitantly. However, influenza vaccination should not be delayed if a specific vaccine is not available.”^12^ Consistent with our study’s safety hypothesis, we found that the proportion of participants with at least 1 severe (grade 3) solicited reactogenicity event was noninferior (not higher) in the simultaneous RZV dose 1 and aIIV4 group (11.5%) compared with the simultaneous RZV dose 1 and HD-IIV4 group (12.5%). The proportion of participants with at least 1 severe (grade 3) solicited local or systemic reactogenicity event evaluated separately was also noninferior (not higher) in the aIIV4 group vs the HD-IIV4 group. No reactions led to a medical visit.

Few participants in our study had SAEs, and the clinical conditions seen after vaccination were those expected in an older adult population. There were 6 SAEs (1 in the aIIV4 group) within 43 days after vaccination with RZV dose 1 and both influenza vaccines, with a small and not statistically significant imbalance between the groups. Nine SAEs were observed during the entire study period, and the imbalance did not persist: 4 of 130 (3.1%) in the RZV and aIIV4 group and 5 of 137 (3.7%) in the RZV and HD-IIV4 group. Study investigators assessed 1 SAE as possibly related to vaccination in the simultaneous RZV dose 1 and HD-IIV4 group. The participant developed a left third cranial nerve palsy 25 days after vaccination, which required a 1-day hospitalization. The palsy resolved completely by 8 weeks after the hospitalization. This SAE was also considered as the sole potentially immune-mediated event identified in the study.

Safety profiles of aIIV4, HD-IIV4, and RZV in our study were consistent with those found in prelicensure studies for each vaccine. For example, the proportions of individuals with any severe reaction was 1.1% of aIIV4 recipients,^3^ less than 1% of HD-IIV4 recipients,^10^ and 11% of RZV recipients aged 70 years or older in prelicensure studies^5^ compared with 11.5% to 12.5% for our study’s simultaneous vaccination with RZV dose 1 and aIIV4 or IIV4-HD, respectively. Our findings were also consistent with a prior CISA study showing similar safety profiles for trivalent formulations of aIIV3 vs HD-IIV3 among older adults^19^ and with a study assessing the safety of RZV and a nonadjuvanted IIV4 administered at the same visit.^20^ There are no studies, and therefore uncertainty, of the safety and reactogenicity of simultaneous vs sequential administration of RZV with adjuvanted or high-dose influenza vaccine.

We observed no differences between groups in the effect of reactogenicity on HRQOL. We observed a decrease in mean index and VAS scores among participants with grade 3 reactions by day 2 that recovered to baseline by days 3 and 4. This same pattern of a decrease and recovery in EQ-5D-5L Utility Index Score and EQ-VAS scores was observed among older adults with grade 3 reactogenicity after RZV.^21,22^

### Limitations

This study is subject to limitations. The study fell short of the enrollment goal due to the COVID-19 pandemic and season 1 start-up logistical issues, which resulted in an underpowered study if our initial assumptions for the null hypothesis were correct. An underpowered study may potentially lead to either a type I or type II error.^23^ The number of participants from Black and Hispanic populations was small, so generalizability of the results is limited to White individuals. The study population consisted of community-dwelling older adults who had a high, self-rated level of health. In line with other randomized clinical trials, the number of participants in the study was too small to detect rare SAEs. Vaccine safety active surveillance systems could be used to assess the frequency of rare safety events, including immune-mediated disease, among older adults receiving aIIV and RZV.

## Conclusions

In this randomized clinical trial of simultaneous administration of RZV dose 1 and aIIV4 in older adults, the proportion of participants with at least 1 severe reaction was not higher after RZV dose 1 and aIIV4 compared with RZV dose 1 and HD-IIV4. There were no significant differences between the groups in the occurrence of SAEs after RZV dose 1 and adjuvanted or nonadjuvanted influenza vaccine. The postvaccination effect on HRQOL was similar between the 2 groups. From a safety standpoint, the simultaneous administration of RZV and aIIV4 was an acceptable option for vaccine delivery among older adults.

## Notes

Supplement 1.
Trial Protocol and Statistical Analysis Plan

Supplement 2. **eMethods 1.** Eligibility Criteria **eTable 1.** Reactogenicity Assessment **eMethods 2.** EQ-5D-5L and VAS **eTable 2.** Local and Systemic Reactions During Days 1 Through 8 Following RZV Dose 1 and aIIV4 vs RZV Dose 1 and HD-IIV4 in Older Adults Aged ≥65 Years **eTable 3.** Proportion of Participants With at Least One Severe (Grade 3) Solicited Local or Systemic Reactogenicity Event on Days 1-8 After RZV Dose 2 in the RZV and aIIV4 Group vs RZV and HD-IIV4 Group **eTable 4.** Proportion of Participants With Moderate-to-Severe (Grade 2-3) Solicited Local or Systemic Reactogenicity Events on Days 1-8 After RZV Dose 1 and After RZV Dose 2 in the RZV and aIIV4 Group vs RZV and HD-IIV4 Group **eTable 5.** Proportion of Participants With at Least One Severe (Grade 3) Solicited Local or Systemic Reactogenicity Event on Days 1-8 After RZV Dose 1 in Each Study Group by Age, 65-69 or ≥70 Years **eFigure 1.** Changes in Health-Related Quality of Life as Measured by EQ-5D-5L Index After RZV Dose 1 and aIIV4 With RZV Dose 1 and HD-IIV4 **eFigure 2.** Changes in Health-Related Quality of Life as Measured by EQ Visual Analogue Scale (VAS) After RZV Dose 1 and aIIV4 With RZV Dose 1 and HD-IIV4

Supplement 3.
Data Sharing Statement
