# Supplementary material for: Safety of Simultaneous Vaccination With Adjuvanted Zoster Vaccine and Adjuvanted Influenza Vaccine: A Randomized Clinical Trial
Source: JAMA Netw Open. 2024 Oct 24;7(10):e2440817. doi: 10.1001/jamanetworkopen.2024.40817 (PMC11581605; doi:10.1001/jamanetworkopen.2024.40817)
Supplement: Supplement 1. — Trial Protocol and Statistical Analysis Plan [file jamanetwopen-e2440817-s001.pdf]

Safety of Simultaneous Vaccination with Zoster Vaccine Recombinant (RZV) and Quadrivalent Adjuvanted Inactivated Influenza Vaccine (aIIV4) (Lead)

Short Title: Simultaneous RZV and aIIV4 Vaccination (Lead)

Centers for Disease Control & Prevention
Clinical Immunization Safety Assessment (CISA) Project

Lead Site (Duke University) Principal Investigator: Ken Schmader, MD
Lead Site Sub-principal Investigator: Emmanuel B. Walter, MD, MPH

Contributing Site (Johns Hopkins University) Investigator: Kawsar Talaat, MD
Contributing Site Sub-principal Investigator: Sean Leng, MD

Centers for Disease Control and Prevention (CDC) Principal Investigator: Karen R. Broder, MD
CDC Sub-principal Investigator: Mike McNeil, MD, MPH

NCT # NCT05007041

Version Number: 7.0

November 27,2023

STATEMENT OF COMPLIANCE

- This trial will be conducted in compliance with the protocol, the International Conference on Harmonization (ICH) Guideline E6—Good Clinical Practice (GCP), and the applicable guidelines and regulatory requirements from the United States (US) Code of Federal Regulations (CFR), 45 CFR Part 46.
- All study personnel with subject contact have completed Human Subjects Protection Training.

TABLE OF CONTENTS

PROTOCOL SUMMARY 5
1 BACKGROUND 10
1.1 Background 10
1.2 Summary and Rationale 12
2 STUDY OBJECTIVES AND OUTCOME MEASURES 13
2.1 Study Objectives 13
2.1.1 Primary Objectives: 13
2.1.2 Secondary Objectives 13
2.1.3 Exploratory Objectives: 13
2.2 Study Outcome Measures 14
2.2.1 Primary Outcome Measures: 14
2.2.2 Secondary Outcome Measures 14
2.2.3 Exploratory Outcome Measures 14
3 STUDY DESIGN 15
3.1 Main study design 15
3.2 Laboratory Studies 15
4 STUDY ENROLLMENT AND WITHDRAWAL 15
4.1 Subject Inclusion Criteria 15
4.2 Subject Exclusion Criteria 16
4.3 Recruitment 17
4.4 Reasons for and Handling of Withdrawals 18
4.5 Termination of Study 18
5 STUDY SCHEDULE, PROCEDURES, & EVALUATIONS 18
5.1 Schedule of events and data collection 18
5.2 Treatment Assignment Procedures 22
5.2.1 Randomization 22
5.3 Data Collection 23
5.3.1 Vaccine Supply, Storage, Administration, and Blinding 23
5.4 Reactogenicity and Safety Assessment 23
5.4.1 Adverse events of clinical interest 26
5.4.2 Causality (relatedness) Assessment 26
5.4.3 Reporting of Adverse Events 26
5.4.4 Safety Monitoring Plan 27
5.4.5 EQ-5D and EQ VAS Scale 27
5.5 Health Care Utilization 28
5.6 Biospecimens Collection & Handling 28
5.6.1 Serum 28
6 LABORATORY ANALYSES 29

6.1 Influenza Hemagglutination Inhibition (HAI) Assay 29
7 STATISTICAL CONSIDERATIONS....29
7.1 Analysis Plan....29
7.1.1 Sample Size 30
7.1.2 Analysis Populations 30
7.1.3 Primary Objective 1 30
7.1.4 Secondary and Exploratory Objectives 31
7.2 Data Management....31
7.2.1 Research Electronic Data Capture (REDCap) 32
7.3 Role of the CDC Investigators in the Project....33
8 HUMAN SUBJECTS 33
8.1 Human Subjects Involvement, Characteristics, and Design....33
8.2 Sources of Material 33
8.3 Potential Risks and Benefits....34
8.4 Adequacy of Protection Against Risks 35
8.4.1 Protections against Risk....35
8.4.2 ClinicalTrials.gov Requirements....35
8.5 Human Subjects....35
8.5.1 Vulnerable Subjects Research 35
REFERENCES 37
APPENDIX....39

PROTOCOL SUMMARY

**Title:**	Safety of Simultaneous Vaccination with Zoster Vaccine Recombinant (RZV) and Quadrivalent Adjuvanted Inactivated Influenza Vaccine (aIIV4) (Lead)
**Phase:**	Phase IV
**Population:**	At least 400 community-dwelling adults ≥65 years of age who intend to simultaneously receive the RZV and aIIV4 or high-dose inactivated influenza vaccine (HD-IIV4) during the 2021-2022 (Year 1) or 2022-2023 (Year 2) influenza seasons.
**Clinical Sites:**	Two: Duke University (Lead); Johns Hopkins University (Contributing)
**Study Duration:**	36 months
**Participant Duration:**	Approximately 3.5 months
**Description of Study Procedures:**	This is a prospective, randomized, blinded clinical trial to assess the safety of simultaneous RZV and aIIV4 versus simultaneous RZV and HD-IIV4 in 400 adults age ≥65 years. Participants aged 65 to 69 will be randomized (1:1) to receive either simultaneous RZV/aIIV4 or RZV /HD-IIV4 using a permuted block randomization scheme stratified by Lead and Contributing Site. A separate permuted block, stratified by site, will be allocated for subjects who are age 70 or older.<br><br>Vaccine reactogenicity will be assessed for 7 days post-injection and compared between the two groups. Serious adverse events and adverse events of clinical interest will be assessed through 42 days post-vaccination and compared between the two groups.<br><br>Detailed health and demographic data will be collected from study participants at baseline prior to influenza vaccine receipt. With Day 1 serving as the day of vaccination, participants will be followed through Day 8 (total 8 days) for symptoms of reactogenicity. Participants will be followed through Day 43 for adverse events, and through the entire period of enrollment for serious adverse events and adverse events of clinical interest.<br><br>Vaccine strain-specific serum hemagglutination inhibition

	antibody titers for each of the 4 influenza strains for the respective seasons will be measured from blood samples collected pre- and 28 days post-immunization for all specimens obtained during year 1 and year 2 of the study period.
**Objectives:**	Primary Objectives:<br>1. To compare the proportion of participants with at least one severe (Grade 3) solicited local or systemic reactogenicity event after RZV dose 1 in the RZV and aIIV4 group versus RZV and HD-IIV4 group.<br>a. Hypothesis: the proportion of participants with at least one severe (Grade 3) solicited reactogenicity event will be noninferior (not higher) in the RZV and aIIV4 group compared with the RZV and HD-IIV4 group.<br><br>Secondary Objectives<br>1. To compare the proportion of participants with at least one severe (Grade 3) solicited local reactogenicity event after RZV dose 1 in the RZV and aIIV4 group vs. RZV dose 1 and HD-IIV4 group<br>2. To compare the proportion of participants with at least one severe (Grade 3) solicited systemic reactogenicity event after RZV dose 1 in the RZV and aIIV4 group vs. RZV dose 1 and HD-IIV4 group<br>3. To compare the proportion of participants with at least one serious adverse event or adverse event of clinical interest after RZV dose 1 in the RZV and aIIV4 group vs. RZV dose 1 and HD-IIV4 group through Day 43 and describe these events<br><br>Exploratory Objectives:<br>1. To compare the proportion of participants with at least one severe (Grade 3) solicited local or systemic reactogenicity event after RZV dose 2 in the RZV and aIIV4 group vs. RZV and HD-IIV4 group<br>2. To compare the proportion of participants with at least one severe (Grade 3) solicited local reactogenicity event after RZV dose 2 in the RZV and aIIV4 group vs. RZV and HD-IIV4 group<br>3. To compare the proportion of participants with at least

	one severe (Grade 3) solicited systemic reactogenicity event after RZV dose 2 in the RZV and aIIV4 group vs. RZV and HD-IIV4 group<br>4. To compare the proportion of participants with at least one serious adverse event or adverse event of clinical interest after RZV in the RZV and aIIV4 group vs. RZV and HD-IIV4 group and describe these events through the entire study period<br>5. To compare the proportion of participants with moderate-to-severe (Grade 2-3) solicited local or systemic reactogenicity event after RZV dose 1 in the RZV and aIIV4 group vs. RZV dose 1 and HD-IIV4 group<br>6. To compare the proportion of participants with moderate-to-severe (Grade 2-3) solicited local or systemic reactogenicity event after RZV dose 2 in the RZV and aIIV4 group vs. RZV and HD-IIV4 group<br>7. To compare serum hemagglutination inhibition (HAI) antibody titers after RZV dose 1 and HD-IIV4 with RZV dose 1 and aIIV4 for each of the four influenza vaccine strains contained in the respective vaccine for that season in the full study population and by age<br>8. To describe and compare changes in health-related quality of life after RZV dose 1 and aIIV4 with RZV dose 1 and HD-IIV4 in the full study population and by age-group
**Outcome Measures:**	Primary:<br>1. Proportion of participants with at least one severe (grade 3) solicited local or systemic reactogenicity event on days 1-8 after RZV dose 1 in each study group<br><br>Secondary:<br>1. Proportion of participants with at least one severe (grade 3) solicited local reactogenicity event on days 1-8 after RZV dose 1 in each study group<br>2. Proportion of participants with at least one severe (grade 3) solicited systemic reactogenicity event on days 1-8 after RZV dose 1 in each study group<br>3. Proportion of participants with at least one serious

	adverse event or adverse event of clinical interest after RZV dose 1 and clinical description of these events in each study group through Day 43.<br><br>Exploratory:<br>1. Proportion of participants with at least one severe (grade 3) solicited local or systemic reactogenicity event on days 1-8 after RZV dose 2 in each study group.<br>2. Proportion of participants with at least one severe (grade 3) solicited local reactogenicity event on days 1-8 after RZV dose 2 in each study group.<br>3. Proportion of participants with at least one severe (grade 3) solicited systemic reactogenicity event on days 1-8 after RZV dose 2 in each study group.<br>4. Proportion of participants with serious adverse events and adverse events of clinical interest after RZV dose 2 and clinical description of these events in each study group through the entire study period.<br>5. Proportion of participants with at least one moderate-to-severe (Grade 2-3) solicited local or systemic reactogenicity event on days 1-8 after RZV dose 1 in each study group.<br>6. Proportion of participants with at least one moderate-to-severe (Grade 2-3) solicited local or systemic reactogenicity event on days 1-8 after RZV dose 2 in each study group.<br>7. HAI titers by vaccination group and age group.<br>a. The proportion of subjects achieving seroconversion at day 29 (an HAI titer ≥ 1:40 at day 29 if the baseline titer is < 1:10 or a minimum four-fold rise in HAI titer if the baseline titer is ≥ 1:10) for each influenza strain in the respective season's vaccine<br>b. Proportion of subjects with a seroprotective HAI titer (≥ 1:40) pre- and post-immunization at day 29 for each IIV antigen in the respective season's vaccine<br>c. The geometric mean HAI titer (GMT) for each IIV antigen in the respective season's vaccine<br>d. The geometric mean fold rise (GMFR) in HAI

	titer for each IIV antigen in the respective season's vaccine<br>8. Change in scores on the EuroQOL 5 dimensions-5 level (EQ-5D-5L) and EuroQOL visual analogue scale (EQ VAS) pre-vaccination and post-vaccination will be compared between the vaccination groups and age groups.
**Estimated Time to Complete Enrollment:**	Approximately 16 months for enrollment during each of two consecutive flu seasons

1 BACKGROUND

1.1 Background

Novel adjuvants

Novel adjuvants are powerful immune stimulants employed in vaccine platforms to improve immunogenicity and efficacy (1). In recent years, the FDA licensed several vaccines with novel adjuvants. More vaccines with novel adjuvants are in pre-clinical development and in clinical trials (2). Clinicians may opt to administer these vaccines simultaneously. Vaccines with novel adjuvants are more reactogenic than vaccines without adjuvants and there is a theoretical possibility that novel adjuvants could activate immune- mediated disease in some individuals (3). In addition, one investigational COVID-19 vaccine uses recombinant spike protein along with a saponin-based Matrix-M adjuvant. Therefore, data are needed on the safety of the simultaneous administration of vaccines with novel adjuvants.

Table 1. FDA Licensed Vaccine Containing Novel Adjuvants

Year Licensed	Novel Adjuvant	Composition	Vaccines
2009	AS04	Monophosphoryl lipid A (MPL) + aluminum salt	Human papillomavirus vaccine, recombinant (Cervarix)
2015	MF59	Oil in water emulsion composed of squalene	Trivalent adjuvanted inactivated influenza vaccine (aIIV3, FLUAD Trivalent)
2017	CpG 1018	Cytosine phosphoguanine (CpG),	Hepatitis B Vaccine, Recombinant, Adjuvanted (Heplisav-B)
2017	AS01B	Monophosphoryl lipid A (MPL) and QS-21	Zoster Vaccine Recombinant, Adjuvanted (Shingrix)
2020	MF59	Oil in water emulsion composed of squalene	Quadrivalent adjuvanted inactivated influenza vaccine (aIIV4 FLUAD Quadrivalent)

<u>Zoster vaccine</u>

For older adults, the need for data on simultaneous administration of vaccines with novel adjuvants has specific clinical relevance for the prevention of influenza and herpes zoster. Herpes zoster is a painful neurocutaneous disease caused by the reactivation of latent varicella zoster virus (VZV) from dorsal root or cranial nerve ganglia (9). The incidence of herpes zoster and chronic pain following zoster, known as postherpetic neuralgia, increases markedly with aging (4). A live attenuated zoster vaccine (Zoster Vaccine Live, ZVL; Zostavax, Merck and Co., Inc) was licensed in 2006 and recommended by the Advisory Committee on Immunization Practices (ACIP) for the prevention of herpes zoster in immunocompetent adults aged ≥50 years old (5). An adjuvanted recombinant zoster

vaccine (RZV; Shingrix, GlaxoSmithKline) was licensed in 2017 and recommended by the ACIP for the prevention of HZ in immunocompetent adults aged ≥50 years (6). Due to the superior efficacy of RZV compared to ZVL, ZVL is no longer sold in the U.S RZV contains the novel adjuvant AS01B and recombinant glycoprotein E (7). AS01B is composed of monophosphoryl lipid A (MPL) from *Salmonella minnesota* and QS- 21, a saponin purified from plant extract *Quillaja saponaria* Molina, combined in a liposomal formulation (7). RZV is administered as a 2-dose series intramuscularly 2-6 months apart (7).

In the ZOE-50 and ZOE-70 phase III clinical trials of RZV versus placebo, 17% of vaccine recipients ≥50 years old and 12% of recipients ≥70 years old reported any grade 3 adverse event (reactions related to vaccination that prevented normal activities) (8, 9). Grade 3 injection-site reactions (pain, redness, and swelling) were reported by 9.4% of vaccine recipients and grade 3 solicited systemic events (myalgia, fatigue, headache, shivering, fever, and gastrointestinal symptoms) were reported by 10.8% of vaccine recipients (8, 9). The proportion of grade 3 reactions decreased with increasing age. The rates of serious adverse events and potential immune-mediated disease over the study periods were similar in the RZV and placebo groups (8, 9).

<u>Influenza vaccine</u>

Influenza is a serious acute viral respiratory illness in older adults caused by influenza A or B viruses. Older adults are at high risk for influenza-related morbidity and mortality due to immunosenescence and age-related diseases (10-12). For persons aged ≥65 years, the ACIP recommends any age-appropriate IIV formulation (standard dose or high dose, trivalent or quadrivalent, unadjuvanted or adjuvanted) or RIV4 as acceptable options (13). Influenza vaccines licensed by the FDA specifically for use in adults aged ≥65 years include unadjuvanted high-dose inactivated influenza vaccines and adjuvanted inactivated influenza vaccines which were developed to improve immunogenicity and efficacy in older adults compared to standard dose vaccines (13). Unadjuvanted trivalent high-dose inactivated influenza vaccine (HD-IIV3; Fluzone® High-Dose; Sanofi Pasteur), licensed in 2010, and unadjuvanted quadrivalent high-dose inactivated influenza vaccine, licensed in 2019 (HD-IIV4; Fluzone® High-Dose Quadrivalent; Sanofi Pasteur) contain four times the amount of hemagglutinin antigen compared with standard dose unadjuvanted inactivated influenza vaccines (14, 15).

Trivalent adjuvanted inactivated influenza vaccine [aIIV3; FLUAD; Seqirus Inc.], and Quadrivalent adjuvanted inactivated influenza vaccine [aIIV4; FLUAD® Quadrivalent, Seqirus Inc.], contain MF59, a -squalene--based adjuvant (14, 16).

In studies that evaluated the safety of aIIV3 and HD-IIV3 versus trivalent, standard dose, unadjuvanted, inactivated influenza vaccine (SD-IIV3), aIIV3 and HD-IIV3 had higher rates of moderate-to-severe reactions compared with SD-IIV3 (16, 17) but grade 3 adverse events were reported by <2% of participants for each solicited local and systemic reaction (16, 17). Rates of serious adverse events were similar in the aIIV3 versus SD-IIV3 groups and in the HD-IIV3 versus the SD-IIV3 groups. A CDC Clinical Immunization Safety Assessment (CISA) Project prospective, randomized clinical trial found that moderate-severe injection-site pain was not higher after aIIV3 compared with HD-IIV3 in adults aged ≥65 years (18), (ClinicalTrials.gov Identifier: NCT03183908). The rates of serious adverse events over the study periods were similar in the aIIV3 and HD-IIV3 groups (18).

1.2 Summary and Rationale

Study Rationale

In clinical practice, the use of RZV has replaced the use of ZVL in older adults (19). The use of high-dose and adjuvanted influenza vaccines is increasingly more common than use of standard-dose unadjuvanted influenza vaccine in adults ≥65 years (20). The simultaneous administration of RZV and either high-dose and adjuvanted influenza vaccines at the same healthcare visit is more convenient for patients and providers and increases the probability that individuals will be fully vaccinated. Unpublished data from the Centers for Medicare and Medicaid Services suggest that simultaneous administration of RZV and aIIV3 is already occurring in some adults aged ≥65 years.

The ACIP General Best Practices Guidelines for Immunization does not address simultaneous administration of vaccines with novel adjuvants (21). A randomized clinical trial to evaluate the safety of simultaneous administration of RZV and a quadrivalent standard-dose unadjuvanted inactivated influenza vaccine (SD-IIV4) observed no safety concerns with either vaccine (22). However, there are no data on the safety of the simultaneous administration of RZV and aIIV3 or aIIV4. Simultaneous administration of RZV and aIIV4 is not labeled as a contraindication (7, 16). Because of lack of data on the safety of simultaneous administration of aIIV3 or aIIV4 with other vaccines containing novel adjuvants and availability of non-adjuvanted influenza vaccines, ACIP Influenza vaccine recommendations for the 2019-2020 season state: “selection of a nonadjuvanted influenza vaccine may be considered in situations in which influenza vaccine and another vaccine containing a novel adjuvant are to be administered concomitantly” (13).

Because RZV and aIIV4 contain different novel adjuvants and will be simultaneously administered by clinicians in this study, it is important to evaluate if simultaneous administration of both vaccines increases the risk for severe reactogenic events compared with simultaneous administration with RZV with a non-adjuvanted vaccine. HD-IIV4 was selected as the unadjuvanted comparator influenza vaccine as the use of HD-IIV has increasingly become the standard of care. In addition, prospectively collecting detailed clinical information about other adverse events such as immune-mediated diseases after simultaneous vaccination with RZV and aIIV4 could also help advance understanding of safety of simultaneous use of vaccines with novel adjuvants in adults. We propose conducting a randomized clinical trial to evaluate the safety of simultaneous administration of RZV (dose 1) and aIIV4 among older adults. A randomized controlled trial is the strongest design to fill these knowledge gaps and address the limitations of observational data collected from the Vaccine Adverse Event Reporting System (VAERS) and Vaccine Safety Datalink (VSD). The information gained from this trial will be novel in that it will likely be the first in human study. It will lead to a better understanding of the safety of the simultaneous administration of these specific vaccines specifically and novel adjuvants in general. These findings are important to the health of older adults and to potentially more efficient vaccine delivery.

Summary

To the best of our knowledge, this is the first study in humans to assess the safety of simultaneous administration of vaccines with novel adjuvants. Findings from this study may advance the health of older adults. By gaining more information about the safety of simultaneous vaccination in older adults, we may reduce the number of health care visits and avoid vaccination delays, which is especially important if SARSCoV-2 (COVID-19) continues to circulate. This information may also be useful if a SARS-CoV-2 vaccine using a novel vaccine construct is introduced in the future.

2 STUDY OBJECTIVES AND OUTCOME MEASURES

2.1 Study Objectives

2.1.1 Primary Objectives:

1. To compare the proportion of participants with at least one severe (Grade 3) solicited local or systemic reactogenicity event after RZV dose 1 in the RZV and aIIV4 group vs. RZV and HD-IIV4 group.
    a. Hypothesis: The proportion of participants with at least one severe (Grade 3) solicited reactogenicity event after RZV dose 1 will be noninferior (not higher) in the RZV and aIIV4 group compared with the RZV and HD-IIV4 group

2.1.2 Secondary Objectives

1. To compare the proportion of participants with at least one severe (Grade 3) solicited local reactogenicity event after RZV dose 1 in the RZV and aIIV4 group vs. RZV and HD-IIV4 group
2. To compare the proportion of participants with at least one severe (Grade 3) solicited systemic reactogenicity event after RZV dose 1 in the RZV and aIIV4 group vs. RZV and HD-IIV4 group
3. To compare the proportion of participants with at least one serious adverse event after RZV dose 1 in the RZV and aIIV4 group vs. RZV and HD-IIV4 group through Day 43 and describe these events

2.1.3 Exploratory Objectives:

1. To compare the proportion of participants with at least one severe (Grade 3) solicited local or systemic reactogenicity event after RZV dose 2 in the RZV and aIIV4 group vs. RZV and HD-IIV4 group
2. To compare the proportion of participants with at least one severe (Grade 3) solicited local reactogenicity event after RZV dose 2 in the RZV and aIIV4 group vs. RZV and HD-IIV4 group
3. To compare the proportion of participants with at least one severe (Grade 3) solicited systemic reactogenicity event after RZV dose 2 in the RZV and aIIV4 group vs. RZV and HD-IIV4 group

4. To compare the proportion of participants with at least one serious adverse event or adverse event of clinical interest after RZV dose 2 in the RZV and aIIV4 group vs. RZV and HD-IIV4 group and describe these events through the entire study period
5. To compare the proportion of participants with moderate-to-severe (Grade 2-3) solicited local or systemic reactogenicity event after RZV dose 1 in the RZV and aIIV4 group vs. RZV dose 1 and HD-IIV4 group
6. To compare the proportion of participants with moderate-to-severe (Grade 2-3) solicited local or systemic reactogenicity event after RZV dose 2 in the RZV and aIIV4 group vs. RZV and HD-IIV4 group
7. To compare serum hemagglutination inhibition (HAI) antibody titers after RZV dose 1 and HD-IIV4 with RZV dose 1 and aIIv4 for each of the four influenza vaccine strains contained in the respective vaccine for that season in the full study population and by age
8. To describe and compare changes in health-related quality of life after RZV dose 1 and aIIV4 with RZV dose 1 and HD-IIV4 in the full study population and by age group

2.2 Study Outcome Measures

2.2.1 Primary Outcome Measures:

1. Comparison of the proportion of subjects reporting at least one severe (grade 3) solicited local or systemic reactogenicity event on days 1-8 after RZV dose 1 in each study group

2.2.2 Secondary Outcome Measures

1. Proportion of participants with at least one severe (grade 3) solicited local reactogenicity event on days 1-8 after RZV dose 1 in each study group
2. Proportion of participants with at least one severe (grade 3) solicited systemic reactogenicity event on days 1-8 after RZV dose 1 in each study group
3. Proportion of participants with at least one serious adverse event or adverse event of clinical interest after RZV dose 1 and clinical description of these events in each study group through Day 43

2.2.3 Exploratory Outcome Measures

1. Proportion of participants with at least one severe (grade 3) solicited local or systemic reactogenicity event on days 1-8 after RZV dose 2 in each study group
2. Proportion of participants with at least one severe (grade 3) solicited local reactogenicity event on days 1-8 after RZV dose 2 in each study group
3. Proportion of participants with at least one severe (grade 3) solicited systemic reactogenicity event on days 1-8 after RZV dose 2 in each study group
4. Proportion of participants with serious adverse events and adverse events of clinical interest after RZV dose 2 and clinical description of these events in each study group through the entire study period
5. Proportion of participants with at least one moderate-to-severe (Grade 2-3) solicited local or systemic reactogenicity event on days 1-8 after RZV dose 1 in each study group

6. Proportion of participants with at least one moderate-to-severe (Grade 2-3) solicited local or systemic reactogenicity event on days 1-8 after RZV dose 2 in each study group
7. HAI titers by vaccination group and age group:
    a. The proportion of subjects achieving seroconversion at day 29 (an HAI titer ≥ 1:40 at day 29 if the baseline titer is < 1:10 or a minimum four-fold rise in HAI titer if the baseline titer is ≥ 1:10) for each influenza strain in the respective season's vaccine
    b. Proportion of subjects with a seroprotective HAI titer (≥ 1:40) pre- and post-immunization at day 29 for each IIV antigen in the respective season's vaccine
    c. The geometric mean HAI titer (GMT) for each IIV antigen in the respective season's vaccine
    d. The geometric mean fold rise (GMFR) in HAI titer for each IIV antigen in the respective season's vaccine
8. Change in scores on the EuroQOL 5 dimensions-5 level (EQ-5D-5L) and EuroQOL visual analogue scale (EQ VAS) pre-vaccination and post-vaccination will be compared between the vaccination groups and age groups

3 STUDY DESIGN

3.1 Main study design

This study is a prospective, randomized, blinded clinical trial to assess the safety of simultaneous RZV and aIIV4 versus simultaneous RZV and HD-IIV4 in 400 adults age ≥65 years. Older adults who have not received IIV during the 2021-2022 and 2022-2023 influenza seasons will be enrolled. Detailed health and demographic data will be collected from study participants at baseline prior to influenza vaccine receipt. With Day 1 serving as the day of vaccination, participants will be followed through Day 8 (total 8 days) for symptoms of reactogenicity as described in **Section 5.4**. Vaccinations will be performed by unblinded study staff with all follow-up assessments being conducted by blinded staff. Study subjects will be blinded throughout the study. Participants will be followed through Day 43 after each dose of RZV for adverse events and adverse events of clinical interest, including health care utilization, and through the entire period of enrollment for serious adverse events and immune-mediated diseases, as described in **Section 5.4 and 5.5**.

3.2 Laboratory Studies

3.2.1 Influenza Hemagglutination Inhibition Assay

Participants will have blood draws on Day 1 (before vaccination) and Day 28 to be stored for serum hemagglutination inhibition (HAI) antibody titers. If funding is available, HAI antibody titers will be compared between groups receiving aIIV4 or IIV4-HD for each of the four influenza vaccine strains contained in the respective vaccines for that season.

4 STUDY ENROLLMENT AND WITHDRAWAL

4.1 Subject Inclusion Criteria

Subjects who meet all of the following criteria will be eligible to participate in this interventional study.

1. Male or female age ≥ 65 years
2. Intention of receiving IIV and RZV based on ACIP-CDC guidelines
3. Able to speak English
4. Willing to provide written informed consent
5. Living in the community
6. Intention of being available for entire study period and complete all relevant study procedures, including follow-up phone calls and clinic visits.
7. If HIV positive, HIV should be clinically stable.

4.2 Subject Exclusion Criteria

Subjects who meet any of the following criteria will not be eligible to participate in this study:

1. IIV or recombinant influenza vaccine (RIV) receipt during the respective 2021-2022 or 2022-2023 influenza season prior to study enrollment
2. Prior receipt of recombinant zoster vaccine (Shingrix)
3. For non-COVID-19 Vaccines:
    - Receipt of any inactivated vaccine within 2 weeks prior to enrollment in this study
    - Receipt of any live vaccine within 4 weeks prior to enrollment in this study
    - Planning receipt of any non-COVID-19 vaccine during the entire period
4. For COVID-19 Vaccines:
    - Receipt of COVID-19 vaccine within 2 weeks prior to enrollment in this study. For those who have initiated a COVID-19 vaccine series, enrollment is not allowed until 2 weeks after the final dose of a COVID-19 vaccine is completed.
    - Planning receipt of a COVID-19 vaccine within 2 weeks after administration of study influenza and first dose recombinant zoster study vaccines.
5. Have acute illness or exacerbation of chronic illness within 72 hours of study vaccination
6. Hospitalization within the last 30 days for any reason
7. History of febrile illness (> 100.0°F or 37.8°C) within the past 24 hours prior to IIV administration
8. Has immunosuppression as a result of an underlying illness or treatment, or use of chemotherapy or radiation therapy within the preceding 12 months
9. Has an active neoplastic disease (excluding non-melanoma skin cancer or prostate cancer that is stable in the absence of therapy) *Participants with a history of malignancy may be included if, after previous treatment by surgical excision, chemotherapy or radiation therapy, the participant has been observed for a period that in the investigator's estimation provides a reasonable assurance of sustained cure
10. A history of autoimmune disease, that requires immunosuppressive agents or any other chronic medical condition considered clinically significant by the investigator
11. Use of chronic oral or intravenous administration (≥14 days) of immunosuppressive doses of steroids, i.e., prednisone >10 mg per day, immunosuppressants or other immune-modifying drugs within 30 days of starting this study. (Use of topical, nasal, or inhaled steroids is permitted)
12. Thrombocytopenia, bleeding disorder, or anticoagulant use contraindicating intramuscular injection (a daily aspirin may be acceptable)
13. Contraindication to IIV receipt including history of severe allergic reaction after a previous dose of any influenza vaccine; or to a vaccine component, including egg protein

14. Contraindication to RZV including history of a severe allergic reaction to any component of the RZV vaccine (including saponin or polysorbate 80) or to dose 2 of RZV
15. History of Guillain-Barré syndrome
16. History of Hepatitis C or active Hepatitis B
17. Receipt of blood or blood-derived products (including immunoglobulin) within 6 months prior to study vaccination
18. Dementia, any cognitive condition, or substance abuse that could interfere with study compliance
19. Anyone who is already enrolled or plans to enroll in another clinical trial with an investigational product within 28 days of vaccine receipt. Co-enrollment in observational or behavioral intervention studies are allowed at any time while enrollment in a clinical trial involving an investigational product (other than vaccine) may occur after 28 days following vaccine receipt
20. Any condition which, in the opinion of the investigators, may pose a health risk to the subject or interfere with the evaluation of the study objectives
21. Anyone who is a relative of any research study personnel
22. Anyone who is an employee of any research study personnel

4.3 Recruitment

Participants ≥65 years of age will be recruited from several sources at Duke University Medical Center (DUMC) and Johns Hopkins University using varying techniques. Study investigators will enroll at least 220 persons at Duke over two seasons (~110 participants per season) and ~180 persons at Johns Hopkins over two seasons (~90 per season).

The general techniques for how subjects will be recruited include the following: Study staff, including PIs and study nurses, will approach their patients in clinic directly about the study during clinic visits; notify other health care professionals in their health system about the study via letters and flyers for potential referrals; notify potential subjects about the study via study registries and recruitment service programs; letters and talks to senior groups in various venues including Senior Centers and Continuing Care Retirement Communities; advertising in newspapers; flyers posted at senior locations; letters and talks to potential referral sources; and letter campaigns to older adults in the surrounding catchment area. More specific mechanisms to DUMC and JHU are described below.

At DUMC potential subjects will be approached via the following mechanisms: 1) Elderly participants of previous vaccine studies who have agreed to enroll in future studies; 2) Duke Center for Aging Human Subjects Registry, a unique long-standing registry of over 3000 individuals who volunteer for human studies; 3) Advertising in senior citizen newspapers and general newspapers in the Durham, Chapel Hill and Raleigh areas; 4) Letter campaign to households of older adults; 5) Duke Geriatric Evaluation and Treatment Clinic (GET Clinic) and Duke University Health System primary care clinics; 6) Triangle continuing care retirement communities; 7) Duke's "Aging" network of senior centers; senior activities; and referrals from professionals who work with seniors cultivated via the work of the Center for Aging. These sources are likely to be sufficient, but if not, we will utilize other past successful strategies. An additional strategy will be the ability to send targeted notifications to potential participants through MaestroCare for Research tools. This has worked well on recent clinical trials.

At JHU subjects will be approached through 1) Johns Hopkins Frailty Registry, which as of January 2020 had over 1000 older adults enrolled; 2) Physiologic and Molecular Basis of the Syndrome of Frailty study, which contains 405 unique participants all over the age of 65; 3) COVID-19 vaccine trials, which enrolled several hundred healthy elderly individuals in 2020; 4) Database of prior volunteers at the Center for Immunization Research (CIR). If these are not sufficient, the CIR currently employs a variety of methods to recruit study participants for both outpatient and inpatient trials. Current recruitment materials include the CIR and recruitment campaign logos, volunteerism-based photos and the Center's recruitment slogan, "**Bringing Immunity to Every Community**" to build brand recognition and bring awareness to upcoming studies at the Center. Flyers and IRB-approved recruitment scripts are posted to social media (CIR Facebook, Twitter, Instagram) and websites that cater to people who are interested in study participation. For community outreach, we partner with local broadcast, cable television, and OTT (over-the-top) providers to run 15- and 30-second commercials that are IRB-approved under our general screening protocol.

At all sites, potential participants may be approached by phone call, email or text. The study will be reviewed with the participant, and if they are interested, then initial eligibility screening will take place on the phone following an IRB-approved script. A waiver of documentation of consent will be approved from the reviewing IRB in order to carry out these screening activities.

4.4 Reasons for and Handling of Withdrawals

The following may be reason for study withdrawal:

- As deemed necessary by the principal investigator (PI).
- Subject withdrawal of consent.
- Loss to follow-up.
- Subject unable to return for study appointments.
- Termination of the study by the sponsor.

Subjects may withdraw their consent for study participation at any time and for any reason, without penalty. Subjects who withdraw from the study prior to randomization will be replaced. Subjects who withdraw from the study after randomization will not be replaced. Data collected before withdrawal will still be used for analysis.

4.5 Termination of Study

This study may be terminated for safety concerns of the principal investigators from the Lead or Contributing sites, CDC, or participating Institutional Review Boards (IRBs).

5 STUDY SCHEDULE, PROCEDURES, & EVALUATIONS

5.1 Schedule of events and data collection

Persons meeting the proposed eligibility criteria (**Section 4**) will be recruited. Written informed consent (Appendix A) will be obtained from study participants prior to conducting any study procedures. Prescreening may take place over the phone prior to Visit 1. Error! Reference ource not found. describes the schedule of study visits with further details below.

Table 1. Proposed Schedule of Events

Study Visit	Visit 1	Visit 2	Visit 3	Visit 4	Visit 5	Visit 6	Visit 7	*Visit 8[(a)]	Visit 9
**Study Day**	1	3	9	29	43	60	69	90	103
**Visit Window (Days)**	n/a	3-6	9-16	29-34	43-50	60-65	69-76	90-97	103-110
**Type of contact**	Clinic	Reminder (e-mail, Text or phone call)	Phone	Clinic	Phone	Clinic	Phone	Clinic	Phone
Informed consent & Medical Release of Information	X								
Review Eligibility Criteria	X					X			
Cognitive assessment	X								
Demographic information	X								
Health History	X		X	X	X	X	X	X	X
Concomitant medications	X		X	X	X	X	X	X	X
Influenza and Zoster Vaccination History	X								
EQ-5D QoL form	X					X			
EQ-VAS QoL form	X					X			
Vital signs including temperature	X			X		X			
Short physical performance battery	X								
***Venipuncture**	X			X		X[(b)]		X[(b)]	
Randomization	X								
Vaccination with RZV	X					X			
Vaccination with aIIV4 or HD-IIV4	X								
Symptom diary and supplies given to participant (Paper)	X					X			
Complete Symptom Diary form (Paper)[(c)]	X					X			
Assess for any immediate reactogenicity symptoms	X					X			
Obtain solicited adverse events			X				X	X	
Obtain unsolicited adverse events			X	X	X		X	X	X
Obtain serious adverse event information and Adverse events of clinical interest			X	X	X	X	X	X	X

Table 1. Proposed Schedule of Events									
**Study Visit**	**Visit 1**	**Visit 2**	**Visit 3**	**Visit 4**	**Visit 5**	**Visit 6**	**Visit 7**	***Visit 8[a]**	**Visit 9**
**Study Day**	1	3	9	29	43	60	69	90	103
**Visit Window (Days)**	n/a	3-6	9-16	29-34	43-50	60-65	69-76	90-97	103-110
**Type of contact**	Clinic	Reminder (e-mail, Text or phone call)	Phone	Clinic	Phone	Clinic	Phone	Clinic	Phone
Obtain heath care utilization data			X	X	X				

a. *Visit 8 will occur at JHU site only
b. Blood draws on Visit 6 (Day 60) and Visit 8 (Day 90) will occur at JHU site only for future use
c. Symptom diary (solicited local and systemic reactogenicity events) and EQ-5D to be completed by participant on Days 1-8 after vaccination, and days 60-68 after second dose of RZV

Visit 1, Study Day 1 - Screening, Enrollment, and Vaccination (Clinic Visit)

- Obtain written informed consent and release of medical record information
- Review and confirm study eligibility
- Perform cognitive assessment with the Mini-Cog tool. Scores 3-5 will be eligible to continue study participation. Scores 0-2 will be ineligible to continue study participation
- Complete EQ-5D quality of life form Conduct short physical performance battery (SPPB). The Short Physical Performance Battery (SPPB) is an objective assessment tool for evaluating lower extremity functioning in older persons. The battery measures balance, gait speed and leg strength (See Appendix C).
- Obtain information on preferred method of contact for follow-up (telephone or email reminder)
- Obtain demographic and medical history including history of influenza vaccination and zoster vaccination
- Obtain concomitant medication use
- Obtain vital signs including oral temperature, blood pressure, and pulse Obtain 15mL blood sample prior to vaccination for serologic analysis (**Section 5.6**)
- Randomize study participant to RZV / aIIV4 or RZV / HD-IIV4 administration (**Section 5.2.1**)
- Administer assigned study products – Trained, licensed unblinded research staff will administer either RZV / aIIV4 or RZV/ HD-IIV4 as described in (**Section 5.3.1)**. Ensure participants receive RZV and inactivated influenza Vaccine Information Sheets (VIS) during visit.
- Dispense symptom diary, thermometer, and ruler (in order to standardize measurements). Review instructions for use of thermometer, ruler, and memory aid completion.
- Assess for any immediate reactogenicity symptoms ≥ 15 minutes after vaccination
- Confirm date of next appointment

Study Days 1 – 8

Participants complete solicited symptom diary and EQ-5D each day at approximately the same time each day.

Visit 2, Study Day 4 (window Days 3-6) Reminder (phone call or text message)
- Reminder message to complete diary entries.

Visit 3, Study Day 9 (window Days 9 – 16) Phone Call
- Study staff will contact study participants to review the diary data and record any solicited and unsolicited adverse events (AEs), serious AEs (SAEs), new onset of chronic medical conditions, and concomitant medications as described in **Section 5.4.** Participants will be reminded that they will be contacted again after Day 9.
- Record health care utilization data

Visit 4, Study Day 29 (window Days 29 – 34) Clinic Visit
- Record any unsolicited AEs, SAEs, new onset of chronic medical conditions, and concomitant medications
- Record health care utilization data
- Obtain 15mL blood for serologic analysis
- Confirm preferred method of contact for follow-up (telephone or email reminder)

Visit 5, Study Day 43 (window Days 43 – 50) Phone Visit
- Record any unsolicited AEs, SAEs, new onset of immune-mediated diseases, and concomitant medications
- Record health care utilization data

Visit 6, Study Day 60 (window Days 60 – 65) Clinic Visit
- Review and confirm study eligibility
- Complete EQ-5D quality of life form
- Obtain vital signs including oral temperature, blood pressure, and pulse
- Record any SAEs, new onset of chronic medical conditions, and concomitant medications as described in **Section 5.3**
- Administer assigned study products – Trained, licensed research staff will administer second dose of RZV
- Dispense memory aid, thermometer (if needed), and ruler (in order to standardize measurements). Review instructions for use of thermometer, ruler, and memory aid completion.
- Assess for any immediate reactogenicity symptoms ≥ 15 minutes after vaccination
  *Obtain 15mL blood for serologic analysis for future use.
  JHU site only

Study Days 60-68 (second dose of RZV)
Participants complete solicited symptom diary and EQ-5D each day at approximately the same time each day.

Visit 7, Study Day 69 (window Days 69 -76) Phone Visit
- Study staff will contact study participants to review the memory aid data and record any unsolicited and solicited adverse events (AEs), serious AEs (SAEs), new onset of

chronic medical conditions, and concomitant medications as described in **Section 5.4**. Participants will be reminded that they will be contacted again after Day 9.

Visit 8, Study Day 90 (window Days 90 – 97) Clinic Visit
****JHU site only***

- Record any unsolicited AEs SAEs, new onset of immune-mediated diseases, and concomitant medications
- Obtain 15mL blood for serologic analysis for future use.

****JHU site only***

Visit 9, Study Day 103 (window Days 103 -110) Phone Visit

- Record any unsolicited AEs, SAEs, new onset of chronic medical conditions, and concomitant medications

Unscheduled Visits

- Record any solicited (≤ day 8) AEs, unsolicited (≤ day 43) AEs, SAEs, new onset chronic medical conditions, and concomitant medications (throughout period of enrollment)
- Record health care utilization data (≤ day 43)
- Confirm preferred method of contact for follow-up (telephone or email reminder)

End of Study

At the conclusion of the study, the statistician will unblind the vaccine type for each participant. The study staff will contact subjects to inform them of the vaccine they received via letter. Study staff will provide two copies of the letter to the subjects: one copy for the subject's personal records and the second copy will be given to their respective primary care provider. Subjects will be responsible for informing their primary care provider about which vaccine they received. Study staff will send the subjects a separate letter with a plain language summary of the main results of the study when available and thanking them for their participation. The main results will not be provided to participants earlier than public release of the findings.

5.2 Treatment Assignment Procedures

This study is a prospective, randomized, blinded clinical trial involving subjects aged ≥65 years of age who are to receive RZV and aIIV4 or RZV and HD-IIV4 simultaneously.

5.2.1 Randomization

Participants aged 65 to 69 will be randomized (1:1) to receive either simultaneous RZV / aIIV4 or RZV / HD-IIV4 using a permuted block randomization scheme stratified by Lead and Contributing Site. A separate permuted block will be allocated for subjects who are age 70 or older. The project statistician will generate permuted block randomization schemes, which will be uploaded to REDCap. The randomization schedule will not be available to the study staff, so the next randomization allocation will not be known before randomization occurs. Following confirmation of study eligibility criteria during Visit 1, participant randomization will be through REDCap with treatment allocation recorded on the CRF.

In the event that REDCap is unavailable, manual randomization will occur through the use of envelopes. The project statistician will prepare 20 envelopes per participant age group per site (total of 40 per site) that will use the same randomization strategy as the primary scheme embedded in REDCap. When an unblinded team member is informed of the participant's age group, he/she will pull the next envelope in order. In order to capture the allocation per subject, a separate form in REDCap will be used by the unblinded personnel to add the assignment. A log will need to be kept at the site capturing these instances.

5.3 Data Collection

5.3.1 Vaccine Supply, Storage, Administration, and Blinding

In order to ensure adherence to study randomization assignment, licensed RZV, aIIV4 and HD-IIV4 vaccines will be administered as study procedures. Licensed RZV, aIIV4 and HD-IIV4 vaccines (prefilled syringes) will be purchased for study administration and maintained at the Duke and JHU study centers, stored at 2° to 8°C in a research-specific medication refrigerator. While research staff maintain daily temperature logs for the medication refrigeration, it is also monitored 24/7 with alarm activation if out of range. Research staff are notified of any alarm activations and have an on-call system in place to report to the research center for further investigation. Any potentially compromised vaccine will be quarantined for further disposition based on site- specific SOPs, investigator assessment and communication with the respective vaccine manufacturers. Shingrix (GSK), FLUAD™ (Seqirus) and Fluzone High-Dose (Sanofi) will be the designated study products.

Administration ofaIIV4 comprising of intramuscular delivery of 0.5 mL total volume and HD-IIV4 comprising of intramuscular delivery of 0.7mL total volume will be administered in one dose.. Vaccine lot numbers, dose, and site of vaccine administration will be recorded by research staff. aIIV4 and HD-IIV4 will be administered to blinded participants by unblinded licensed research nurses at the Duke and JHU study centers. RZV will be administered in two doses, approximately 60 days apart, comprising of intramuscular delivery of 0.5mL total volume per dose. After administration, used study syringes will be disposed of according to standard operating procedure. Emergency management supplies will be available for initial treatment of an allergic reaction, if needed. Additionally, a blinded post-assessment team will be present to assist study subjects.

5.4 Reactogenicity and Safety Assessment

Participants will be assessed for any immediate reactogenicity in a period at least 15 minutes after vaccine administration while at the study site. They will be assessed for the following potential systemic reactions such as anaphylaxis related symptoms (e.g., dyspnea, chest tightness, wheezing, cough, stridor, urticarial, flushing, nasal congestion, dizziness, syncope, diaphoresis, emesis) or other medical symptoms (e.g., feverishness (chills/shivering/sweating), fatigue, malaise, myalgia, arthralgia, headache, and gastrointestinal symptoms (nausea, vomiting, diarrhea, abdominal pain). They will be assessed for any potential injection site reactions including pain, erythema or induration. Pain severity will be assessed on a 0 – 10 scale with 10 being the worst pain imaginable and 0 being no pain.

Frequency and occurrence of solicited local and systemic reactogenicity will be assessed daily through post- vaccination Day 8 using a standard memory aid. Unsolicited adverse events (AEs) will be assessed daily through post-vaccination Day 42 following each

vaccination visit. Serious AEs (SAEs) and adverse events of clinical interest including new onset of immune-mediated diseases and concomitant medication use will be assessed daily through the entire period of a subject's participation.

At the time of study enrollment, participants will be given a thermometer and instructed on using the symptom diary to document oral temperatures and post-injection symptoms. Beginning on the evening of Study Visit 1 (Day 1) following vaccination, participants will record their oral temperature using the study-supplied thermometer, the occurrence of AEs, and concomitant medication use for the next 8 days (Day 1 – 8). Temperature will be recorded at roughly the same time each day. If a temperature ≥ 100.0°F (37.8°C) is recorded, a second measurement will be taken. If more than one temperature is taken on the same day, the highest temperature should be recorded. Fever will be defined as a measured temperature ≥ 100.0° F (37.8° C). Participants will be queried during Visits 3 and 7 on any solicited adverse events following vaccination and during Visits 3 and 7 on solicited injection site adverse events, which will be classified as mild, moderate, or severe as described by in **Table 2**. Injection site reactogenicity will be assessed separately for IIV4 and RZV.

Table 2. Injection-site Reactogenicity

Symptom	Mild (Grade 1)	Moderate (Grade 2)	Severe (Grade 3)
**Pain**	Any pain neither interfering with nor preventing normal every day activities.	Painful when limb is moved and interferes with every day activities.	Significant pain at rest. Prevents normal every day activities.
**Induration/ Swelling**	≥ 20 mm to ≤ 50 mm diameter	> 50 mm to ≤ 100 mm diameter	> 100 mm diameter
**Erythema (Redness)**	≥ 20 mm to ≤ 50 mm diameter	> 50 mm to ≤ 100 mm diameter	> 100 mm diameter

Participants will also be queried during Visits 3 and 7 on common post-injection systemic symptoms as described in **Table 3**.

Table 3. Systemic Reactogenicity

Systemic	Mild (Grade 1)	Moderate (Grade 2)	Severe (Grade 3)
**Fever (°C)**	≥ 37.5 - < 38.4° C<br>≥ 100.0 - < 101.1° F	≥ 38.4 - < 39° C<br>≥ 101.1 - < 102.2° F	≥ 39° C<br>≥ 102.2° F
**Fatigue/ Malaise**	Fatigue that is easily tolerated	Fatigue that interferes with normal activity	Fatigue that prevents normal activity
**Myalgia**	Myalgia that is easily tolerated	Myalgia that interferes with normal activity	Myalgia that prevents normal activity
**Arthralgia**	Arthralgia that is easily tolerated	Arthralgia that interferes with normal activity	Arthralgia that prevents normal activity
**Headache**	Headache that is easily tolerated	Headache that interferes with normal activity	Headache that prevents normal activity

Table 3. Systemic Reactogenicity			
**Systemic**	**Mild (Grade 1)**	**Moderate (Grade 2)**	**Severe (Grade 3)**
**Gastrointestinal symptoms (nausea, vomiting, diarrhea, and/or abdominal pain)**	Gastrointestinal symptoms that are easily tolerated	Gastrointestinal symptoms that interfere with normal activity	Gastrointestinal symptoms that prevent normal activity
**Chills/Shivering**	Shivering that is easily tolerated	Shivering that interferes with normal activity	Shivering that prevents normal activity

Participants will be encouraged to report any significant adverse and serious adverse events in an open-ended question format, e.g. "How are you doing? Are you having any medical or clinical problems? If so, please tell me about them." Participants who report severe solicited adverse events or express any concern about symptoms/unsolicited events will be encouraged to follow up with their primary care provider. Study staff will assist with coordination of referral appointments as necessary. Medical records will be obtained and reviewed for any serious adverse events or new onset immune-mediated diseases throughout the study period.

RZV, aIIV4 and HD-IIV4 are recommended for routine immunization for older adults such that we do not anticipate having a significant issue with serious adverse events (SAEs). An SAE is defined as an AE that meets one of the following conditions:

- Results in death during the period of protocol-defined surveillance
- Is life-threatening (defined as immediate risk of death at the time of the event)
- Requires inpatient hospitalization during the period of protocol-defined surveillance
- Results in prolongation of an existing hospitalization
- Results in congenital anomaly or birth defect
- Results in a persistent or significant disability/incapacity
- Any other important medical event that may not result in death, be life threatening, or require hospitalization, may be considered an SAE when, based upon appropriate medical judgment, the event may jeopardize the subject and may require medical or surgical intervention to prevent one of the outcomes listed above

SAEs will be reported to the CDC, DUHS, and JHU IRBs within 48 hours of study staff awareness of the event. If indicated, SAEs will be reported through the CDC's VAERS system.

We will also collect information regarding the occurrence of any new onset immune-mediated diseases (Appendix F). We will monitor study participants for the development of new onset immune-mediated diseases throughout the study period.

Table 1 Unsolicited Adverse Events			
**Systemic Illness**	**Mild (Grade 1)**	**Moderate (Grade 2)**	**Severe (Grade 3)**

**Illness or clinical adverse event**	Noticeable with no limitation in normal daily activity	Some limitation in normal daily activity	Completely unable to perform normal daily activity

We will monitor study participants for the development of new onset acute or chronic medical conditions during the protocol-defined surveillance period of 42 days post-vaccination. These AEs will be reviewed periodically by a safety-monitoring panel (**Section 5.4.4**). We will monitor study participants for SAEs during the protocol-defined surveillance period of 42 days post-vaccination.

5.4.1 Adverse Events of Clinical Interest

We will also collect information regarding the occurrence of any adverse events of clinical interest. Adverse events of clinical interest include syncope during post-vaccination monitoring in clinic, anaphylaxis in the first 24 hours after immunization, and new onset immune-mediated disease as defined in Appendix F.

5.4.2 Causality (relatedness) Assessment

Study site investigators will assess relatedness to vaccine or study procedures (related, possibly related, unlikely related, or not related) for SAEs, AEs, and AEs of clinical interest. Relatedness determinations of these events will inform IRB reporting and safety monitoring (**Section 5.4.3**). Solicited symptoms in Error! Reference source not found. will all be considered to be related to accine and causality assessment will not be done for these events. The study investigators will use their clinical judgement to make causality assessments and may consult the Expert Safety Panel or CISA Project for assistance with causality determinations. The final causality assessment decision is the responsibility of the site PI where the subject was enrolled.

5.4.3 Reporting of Adverse Events

An adverse event (AE) is any untoward medical occurrence in a patient or clinical investigation subject administered a pharmaceutical product and which does not necessarily have to have a causal relationship with this treatment.

If indicated, AEs occurring during the study will be reported to the Vaccine Adverse Event Reporting System (VAERS). The National Childhood Vaccine Injury Act requires healthcare providers to report the following AEs to VAERS:

- Any adverse event listed by the vaccine manufacturer as a contraindication to further doses of the vaccine; or
- Any adverse event listed in the VAERS Table of Reportable Events Following Vaccination [PDF - 75KB] that occurs within the specified time period after vaccination.

In addition, CDC encourages reporting of any clinically significant adverse event that occurs in a patient following a vaccination, even if there is uncertainty regarding if a vaccine caused the event.

SAE and AE reporting will occur consistent with institutional policy. The original verbatim terms used by investigators to identify SAEs and adverse events of clinical interest in the case report form will be mapped to preferred terms using the Medical Dictionary for Regulatory Activities (MedDRA) dictionary (http://www.meddra.org/).

Vaccine-related SAEs will be medically attended per routine care.

SAEs will be reported promptly to the overseeing IRBs in accordance with institutional procedures. Any unanticipated problems resulting from study conduct related to participation will be reported promptly to the reviewing IRBs and CDC, in accordance with institutional procedures.

The period for monitoring and reporting SAEs will occur for the duration of study participation (42-days post vaccination)

The study will report only SAEs occurring during each subject's participation in the study.

More information on potential risks and benefits is located under Human Subjects, **Section 8.3**.

5.4.4 Safety Monitoring Plan

Although RZV and aIIV4 are recently licensed vaccines with novel adjuvants, there is no safety experience with these vaccines administered simultaneously. Vaccines with novel adjuvants are more reactogenic than vaccines without adjuvants. Given that these adjuvants are powerful immune stimulants and that clinicians may opt to administer these vaccines simultaneously, data are needed on the safety of the simultaneous administration of vaccines with novel adjuvants.

Therefore, the goal of the safety monitoring plan is to protect the health of the study population and ensure adequate communication of potential risks, and to provide situational awareness of potential safety signals from this study to CDC Immunization Safety Office (ISO) leadership. This plan is designed to monitor safety while minimizing introduction of bias into the study and minimizing burden to study investigators. The safety monitoring plan is described in Appendix G.

5.4.5 EQ-5D and EQ VAS Scale

The EQ-5D is a standardized, generic measure of health status that provides information on health-related quality of life and activities of daily living relevant to older adults: mobility, self-care, usual activities, pain/discomfort and anxiety/depression (http://www.euroqol.org/) (23). In addition, the instrument contains the EQ Visual Analogue Scale (EQ-VAS) which measures the respondent's self-rated health.

The EQ-5D-5L is the new version of the EQ-5D that increases the levels of severity from three to five to significantly increase reliability and sensitivity while maintaining feasibility and reducing ceiling effects (Appendix H) (24, 25).The descriptive system comprises 5 dimensions of mobility, self-care, usual activities, pain/discomfort, anxiety/depression. For each of these dimensions, there are 5 response levels: no problems, slight problems, moderate problems, severe problems, and extreme problems. The respondent is asked to indicate his/her health state by ticking in the box against the most appropriate statement in each of the 5 dimensions. This

decision results in a 1-digit number expressing the level selected for that dimension. The digits for 5 dimensions can be combined in a 5-digit number describing the respondent's health state from 11111 as best health and 55555 as worst health. These numbers are converted to a Utility Index that ranges from -0.109 (worst health) to 1.000 (best health) for US specific values. The minimum clinically important difference ranges from 0.05 to 0.1 depending on health conditions being studied. The EQ VAS records the respondent's self-rated health on a 20 cm vertical, visual analogue scale with endpoints labelled 'the best health you can imagine' (100) and 'the worst health you can imagine' (0). The respondent marks an 'X' on the scale number to indicate how their health is 'today.' The minimum clinically important difference on the VAS is 8.

The EQ-5D-5L, EQ-VAS have several advantages for use in this study. The measure is applicable to a wide range of health conditions and treatments and provide a simple descriptive profile and a single index value for health status. It has been validated in US and international populations and in older adults (23, 26, 27). The measure is useful for monitoring the health status of patient groups at different moments in time and assessing the seriousness of conditions at different moments in time. The measure is designed for self-completion by respondents. It is simple, straightforward, take only a few minutes to complete and can easily be completed by older adults. The instrument was designed to reduce respondent burden while achieving standards of precision for purposes of group comparisons involving multiple health dimensions. It has been widely used throughout the world in many different studies, including randomized controlled clinical trials, vaccine studies, and health-related quality of life studies in older adults.

5.5 Health Care Utilization

Participants will be asked to report health care utilization including: telephone calls to the medical provider for medical advice, e-mail portal, electronic health record, clinic visits, urgent care visits, emergency department visits and hospital admissions occurring through day 43 according to the schedule in Error! Reference source not found. above. The reason for health are use will also be obtained. Electronic or paper health records will be obtained and reviewed to confirm reports of clinic visits, urgent care visits, emergency department visits and hospital admissions. Health care utilization and the reason for health care utilization will be recorded on the symptom diary.

5.6 Biospecimens Collection & Handling

5.6.1 Serum

Blood specimens will be collected during study visits as described in Error! Reference source ot found..

All blood samples (≈15 mL) will be collected into serum separator tubes and processed as follows:

- Allow blood to clot at room temperature for at least 30 minutes while standing upright in a rack.
- Centrifuge tube within 8 hours of collection at 1100 to 1300 RCF(g) for 10 minutes.
    - Gently remove the vacutainer stopper avoiding serum contamination with red blood cells. Using a single-use pipette, transfer 1.0 mL aliquots of serum (top layer) into 1.0mL or 1.8 mL cryovials, up to 5 cryovials are expected. If less than 1 mL of

processed serum is collected, it is a protocol deviation

- All cryovial aliquots will be barcode labelled and contain a unique identifier via REDCap. Numbers should be placed lengthwise on the tube.
- Freeze the cryovials at -80°C in the temperature-monitored research center freezer for future shipment.
- Serum aliquots will be stored in the Duke Human Vaccine Institute Accessioning Lab, and the Johns Hopkins Hospital until planned HAI analyses at which point the samples stored at JHU will be shipped to Duke where all HAI analyses will be done

6 LABORATORY ANALYSES

6.1 Influenza Hemagglutination Inhibition (HAI) Assay

Influenza Hemagglutination Inhibition (HAI) Assays will be performed on sera collected, contingent on additional funding. Briefly, reference wild-type, reassortant, or vaccine virus strains representative of the specific viral antigens included in the 2021-2022 and 2022-2023 influenza vaccine will be used to evaluate the relative levels of all four influenza strain-specific antibodies in participant serum samples collected pre- and 28 days post-vaccination. To accomplish these activities all participant samples will be interrogated for influenza antibodies against the strains of interest using the influenza hemagglutination inhibition assay (HAI). This assay is considered the "gold-standard" measure by which to evaluate seroconversion/seroprotection in response to seasonal influenza vaccination. This assay will be performed in accordance with the Duke Regional Biocontainment Laboratory Virology Unit's fully optimized and approved SOP (RVUSOP004 Influenza HI of Serum Samples). Briefly, test samples will be assayed by HAI as duplicate 2-fold dilution series starting at 1:10. Serum dilutions are then incubated with a concentration of virus verified to possess a known potential for red blood cell (RBC) agglutination. The presence of virus-specific antibodies is visualized via incubation of the virus-serum mixture with a RBC solution; the endpoint titer for a given dilution series is then expressed as the reciprocal of the final dilution in which complete HAI is observed. By convention, seronegative samples are defined as having an endpoint HAI titer < 40 and seropositive samples as having an endpoint titer of ≥1:40; and seroconversion as a 4-fold change in endpoint titer relative to pre-immunization baseline or a change from <10 to ≥1:40 (28).

7 STATISTICAL CONSIDERATIONS

In collaboration with the Johns Hopkins site, the research team at Duke will oversee the statistical analysis. Data will reside on a secure Duke server maintained by Duke Health Technology Solutions (DHTS). For the study, a database will be developed and a data set for the study without personal identifiers will be made available to the CDC upon request. Duke statisticians will develop a comprehensive Statistical Analysis Plan. The summary points of the analysis plan are presented below.

7.1 Analysis Plan

Should an interim safety analysis be required, the alpha level will be adjusted to assure the overall type I error is maintained at the one-sided alpha 0.025 level for the primary outcome of non-inferiority.

7.1.1 Sample Size

Safety: Based on information in from the pre-licensure trials for RZV, we assume that 14% of adults ≥65 years will have at least one severe solicited local or systemic reactogenicity event after RZV dose 1. This assumption is based on information in from the pre-licensure trials for RZV. We have selected a clinically meaningful noninferiority margin of 10%. Statistical calculations, without consideration of drop-out, show that with a one-side alpha level of 0.025, we would need 380 total subjects (190 subjects in each group across all study sites) to have at least 80% power to be able to demonstrate that the proportion of adults ≥65 years with at least one severe solicited local or systemic reactogenicity event after RZV dose 1 is noninferior after RZV and allV4 versus RZV and HD-IIV4. Assuming a 5% drop out rate, we plan to recruit and randomize 400 total subjects.

7.1.2 Analysis Populations

Intention-to-Treat (ITT) Population:
For Secondary Objective 3 and Exploratory Objectives 4 and 9, the primary analysis population will be the ITT Population; defined as all subjects who are randomized and vaccinated.

Modified Intention-to-Treat (mITT) Population:
For Primary Objective 1, Secondary Objectives 1 and 2, and Exploratory Objectives 1, 2, 3, 5 and 6 the primary analysis population will be the mITT Population; defined as all subjects who are randomized, vaccinated, and provide at least one day of complete data on the symptom diary.

Immunogenicity Population:
For Exploratory Objectives 7 and 8 (immunogenicity analyses) the primary analysis population will be the Immunogenicity Population 2; defined as subjects who received vaccine, provide baseline and Visit 4 blood draws of acceptable volume and quality within the protocol-defined time frame with no protocol violations affecting immunogenicity. Protocol violations affecting the immunogenicity analyses will be defined in the Statistical Analysis Plan (SAP).

7.1.3 Primary Objective 1

- To compare the proportion of older adults with at least one severe (Grade 3) solicited local or systemic reactogenicity event after RZV dose 1 in the RZV and aIIV4 group vs. RZV and HD-IIV4 group.

    - Hypothesis: The proportion of participants with at least one severe (Grade 3) solicited reactogenicity event after RZV dose 1 will be noninferior (not higher) in the RZV and aIIV4 group compared with the RZV and HD-IIV4 group

This objective will be assessed using a one-sided noninferiority test with the alpha level set at 0.025 and noninferiority margin of 10%. The null hypothesis is RZV and allV4 is inferior (i.e., RZV and allV4 will have a higher proportion) to RZV and HD-IIV4 in regards to the proportion of adults with at least one severe (Grade 3) solicited local or systemic reactogenicity event after RZV does 1.

Ho: RZV and aIIV4 - RZV and HD-IIV4 ≥ 0.10 (10%)

The alternative hypothesis is RZV and aIIV4 is noninferior to RZV and HD-IIV4 in regards to the proportion of adults with at least one severe (Grade 3) solicited local or systemic reactogenicity event after RZV dose 1.

Ha: RZV and aIIV4 - RZV and HD-IIV4 < 0.10 (10%)

The upper bound of a site-stratified Newcombe binomial confidence interval with Cochran-Mantel-Haenszel (CMH) weighting of the difference will be used to make this assessment (29).

7.1.4 Secondary and Exploratory Objectives

The statistical methodology described above for the Primary Objective will also be applied to all Secondary Objectives 1 and 2 and Exploratory Objectives 1, 2, 3, 5 and 6. For Secondary Objective 3 and Exploratory Objective 4, the proportion of participants with severe adverse events will be presented in a table along with exact 95% confidence intervals. The confidence intervals will be used to make comparisons and interpretations. For Exploratory Objective 7, seroconversion or seroprotection at day 29 will be compared using a Mantel-Haenszel statistic in a stratified analysis by site. HAI titer (GMTs) and the geometric mean fold rise (GMFR) in HAI titer will be compared using a regression model that will control for site or nonparametric testing if normality assumptions are not met.

For Exploratory Objective 8, the EQ-5D-5L responses are converted to a Utility Index that ranges from -0.109 (worst health) to 1.0 (best health) using the US specific value sets (http://www.euroqol.org/fileadmin/user_upload/Documenten/Excel/Crosswalk_5L/EQ-5D-5L_Crosswalk_Value_Sets.xls). The EQ VAS has a range of 0 (worst health) to 100 (best health). The changes from baseline will be assessed within vaccine group using a paired t-test for the index values and VAS. If normality assumptions are not met, the testing will be performed with a Wilcoxon signed-rank test. The Mann-Whitney U test (Wilcoxon rank-sum test) will be used to compare the difference scores from baseline between the two vaccine groups for the index values and VAS.

Proportions for all objectives will be presented in tables and the noninferiority comparisons will be presented in graphical displays as well. There will be no adjustment to the alpha level for these comparisons, with all noninferiority comparisons at the one-side alpha 0.025 level with a 10% noninferiority margin. The alpha level will be set at the two-side 0.05 level for Exploratory Objectives 7, 8, and 9.

7.2 Data Management

The novel Vanderbilt-designed resource developed specifically for online collection of research information, the Research Electronic Data Capture (REDCap) platform (https://projectredcap.org/), will be used to design study forms, including the reaction forms, and short customized questionnaires to collect information from study subjects. REDCap provides: 1) a streamlined process for rapidly building a database; 2) an intuitive interface for collecting data, with data validation and audit trail; 3) automated export procedures for seamless data

downloads to common statistical packages; 4) branching logic, file uploading, and calculated fields; and 5) a quick and easy protocol set-up. This system will be used by Duke for data management. All electronic linkages will fulfill regulations for protection of human subjects and requirements to minimize the risk of breach of confidentiality.

All study-related documents containing protected health information, e.g. enrollment logs, case report forms, diaries (Appendix E) completed by study participants, will be maintained in secure research offices at Duke and Johns Hopkins University, which are accessible to research staff only.

The study team will utilize a secure, encrypted, file transfer method for sharing study documents and data with the CDC. No personal identifiers will be included in any shared documents or datasets.

7.2.1 Research Electronic Data Capture (REDCap)

REDCap (http://project-redcap.org/) assists with the collection and management of data for diverse clinical and translational research studies. REDCap was designed around the concept of giving research teams an easy method to specify project needs and rapidly develop secure, web-based applications for collection, management and sharing of research data. REDCap accomplishes these key functions through use of a single study metadata table referenced by presentation-level operational modules. Based on this abstracted programming model, databases are developed in an efficient manner with little resource investment beyond the creation of a single data dictionary. The concept of metadata-driven application development is well established, and the critical factor for successful data collection lies in creating a simple workflow methodology allowing research teams to autonomously develop study-related metadata in an efficient manner. Both products include secure institutional data hosting and include full audit-trails in compliance with Health Insurance Portability and Accountability Act (HIPAA) security requirements. The REDCap Consortium is comprised of 647 active institutions. The REDCap currently supports 68,000 projects with over 89,000 users spanning numerous research focus areas across the consortium. The current project will use this software application for the design of electronic forms to collect information from study participants, to link the baseline data, sample collection date, and laboratory results in an automated database family, to perform data cleaning and data quality assurance efficiently, and to design an analytical dataset for the analysis of the project data.

Data will be entered into the REDCap database by members of the study team from Duke and Johns Hopkins using the paper case report forms utilized to record data collected as part of study procedures. Study investigators will be responsible for assuring that all paper records are securely stored according to the requirements of their IRBs. The study investigators will be responsible for assuring the accuracy of the data entered from the paper forms into REDCap. Only the assigned identifiers will be used in REDCap. Therefore, personal health identifiers will not appear in the REDCap database.

In order to perform data cleaning and data quality assurance efficiently, numerous built-in filters and checks for consistency of the data including range and limit checks, branching logic and pull

down menus to limit choices for categorical variables to a pre-specified list will be implemented and performed automatically to minimize data entry error. The data will be randomly sampled and checked against source records on a regular basis. The data and related analytical datasets will also be stored at the lead and contributing sites with secured password-protected computers.

7.3 Role of the CDC Investigators in the Project

This study is funded by a CDC contract with Duke University and Johns Hopkins University as Task Orders in the CISA Project Contract. The Duke University PI (Ken Schmader) will oversee the study in partnership with the Johns Hopkins University, PI (Kawsar Talaat). CDC staff will collaborate with both sites to develop the protocol, conduct the study, ensure the study is aligned with US Department of Health and Human Services (CDC) public health priorities, and analyze the data and disseminate the results. CDC may receive access to coded data not containing any directly identifying information.

8 HUMAN SUBJECTS

8.1 Human Subjects Involvement, Characteristics, and Design

Duke and Johns Hopkins University investigators will be responsible for submitting the protocol, informed consent (Appendix A), diaries (Appendix E), recruitment letters (Appendix I), flyers (Appendix J), and any written or verbally conveyed materials (Appendix K) specific to this project to their institutional review boards. CDC staff will be responsible for submitting materials to the CDC for Human Subjects review and approval.

To facilitate subject recruitment at the practices, we will request a waiver of consent and HIPAA authorization for ascertainment (identification, selection) and/or recruitment of potential subjects while recording identifiable private health information (PHI) prior to obtaining the subject's consent. This information will be obtained from review of the electronic scheduling and medical record systems in the clinics in order to determine eligibility for study enrollment. We will review only the minimum amount of information necessary to determine eligibility, i.e. date of birth, medical and surgical history, and recent laboratory test results. The PHI collected prior to consent will be used to recruit and screen only. Use of PHI in this manner involves no more than minimal risk to subjects and no information will leave the study sites.

Requests for continuing review, when required, will be submitted at each engaged institution in accordance with institutional procedures. Protocol deviations or concerns about study integrity will be reported promptly to the overseeing IRB or CDC in accordance with institutional requirements.

8.2 Sources of Material

Medical history and immunization history will be obtained from the medical record and from patient report. Demographic information will be obtained from the medical record and patient report. Subjects will record solicited adverse reactogenicity events and any medical intervention sought on study days 1-8 on the symptom diary (Appendix E). Diary information will be reported to the study team during a telephone call. The research staff will assess one or more of the following: weight, height, temperature, blood pressure, and pulse.

8.3 Potential Risks and Benefits

RZV, aIIV4, and IIV-HD4 are FDA-licensed vaccines approved for use in adults ≥65 years old. These vaccines are standard clinical practice and recommended by the CDC. Participants will be provided with the CDC Vaccine Information Statement (VIS) for IIV and RZV (https://www.cdc.gov/vaccines/hcp/vis/vis-statements/flu.pdf and https://www.cdc.gov/vaccines/hcp/vis/vis-statements/shingles-recombinant.pdf).

Syncope (fainting) can occur in association with administration of injectable vaccines. Sitting or lying down for about 15 minutes can help prevent fainting, and injuries caused by a fall, as recommended in the ACIP General Recommendations on Immunization(30). Subjects should inform their doctor should they feel dizzy, or have vision changes or ringing in the ears.

IIV risks include minor problems such as soreness, redness, swelling, or pain where the shot was given, hoarseness, sore, red or itchy eyes, cough, fever, aches, headache, itching, fatigue, all of which usually occur within 1-2 days of vaccination and are self-limiting. Some people get severe pain in the shoulder and have difficulty moving the arm where a shot was given. This happens very rarely. More serious problems including a small increased risk of Guillain-Barré Syndrome estimated at 1 or 2 additional cases per million people vaccinated. This is much lower than the risk of severe complications from influenza infection, which can be prevented by IIV(31). In addition, any medication can cause a severe allergic reaction, or anaphylaxis, which is estimated at ~ 1 in one million doses of IIV administered (32).

RZV risks include pain, redness, itching and swelling in the arm where the shot was given. These side effects were usually mild; however, occasional severe local reactions, mainly pain was reported. These local reactions usually went away within 3 to 4 days. Systemic side effects include tiredness (fatigue), muscle pain, headache, chills, fever, and digestive symptoms (nausea, vomiting, diarrhea or stomach ache) and feeling generally unwell (malaise). These symptoms were also usually mild and resolved within few days. About 1 out of 6 people who receive RZV experienced side effects that prevented them from doing regular activities like yardwork or swimming. More serious problems including a small increased risk of Guillain-Barré Syndrome estimated at 3 to 6 additional cases per million people vaccinated. This is much lower than the risk of severe complications from herpes zoster infection, which can be prevented by RZV (33).

Risks of blood drawing include pain, swelling, bleeding, or bruising at the site where the blood sample is collected. Subjects may also experience dizziness or fainting. There is a small risk of infection around the vein where the blood was collected. Each study subject will be asked to have up to 3 blood samplings with the total volume not to exceed 30mL over approximately 6 month period of time at Duke University, and up to 5 blood samplings with a total volume not to exceed 60mL over approximately 6 month period of time at Johns Hopkins University.

As with any licensed vaccine, protection may not occur in 100% of vaccinated persons.

An additional risk of study participation is the potential for loss of confidentiality.

8.4 Adequacy of Protection Against Risks

8.4.1 Protections against Risk

To decrease the possibility of infection at the site of blood drawing, the area on the arm above the vein where blood will be taken will be prepped with 70% isopropyl alcohol antiseptic prior to venipuncture.

Subjects will be counseled on possible side effects following vaccination and followed closely during the 8 days post-vaccination for assessment of moderate to severe local or systemic reactogenicity. Subjects will be evaluated and cared for as described in the Unscheduled Visit section above. All subjects will be monitored in a sitting or lying position for 15 minutes following vaccinations to help prevent fainting, and injuries caused by a fall. Subjects with a prior history of severe allergic reaction after a previous dose of any influenza vaccine, or to a vaccine component, including egg protein, will be excluded from study enrollment. Data Safety monitoring, as described above (**Section 5.4.4** and Appendix G), shall also be done.

The study team will provide documentation to the participant and primary care provider regarding receipt of influenza vaccine without specification of whether it was high dose or adjuvanted vaccine to preserve blinding.

If a participant's care requires the identity of the vaccine received, blinding will be broken for that patient. At the end of the study, the participants and providers will receive documentation about which vaccine the participant received.

Every effort possible will be made to keep information about participants confidential. Computerized participant information will be kept in password-protected files on secured servers. Paper case report forms will be kept in locked files belonging to the study personnel. Any publications resulting from this work will not contain any identifiable participant information.

8.4.2 *ClinicalTrials.gov Requirements*

The project is registered on ClinicalTrials.gov (NCT # NCT05007014)

8.5 Human Subjects

In obtaining and documenting informed consent, the Investigator and study team will comply with the applicable regulatory requirements, Good Clinical Practices, and ethical principles. The written informed consent form must be signed and dated by the study participant prior to initiation of any study activities.

8.5.1 Vulnerable Subjects Research

This study proposes to include subjects with mild cognitive impairment. All potential subjects will undergo cognitive assessment to ensure they are capable of providing consent. Mild cognitive impairment is a common age-related condition that is defined by the presence of short-term memory impairment that does not interfere the individual's ability to perform activities of daily living or affect other areas of cognition, including judgment and independent decision-making. Therefore, persons with mild cognitive impairment have the capacity to make decisions about their health care choices, including influenza vaccination, and participation in research

studies. Influenza vaccination is recommended for these individuals. The benefits and burdens of the proposed study apply equally to these individuals as to persons without mild cognitive impairment.

Potential participants may have low scores on the cognitive screening tests and be ineligible for the study. In this case, the study doctor or designee will review the results with the individual and recommend follow-up with the individual’s health care provider.

REFERENCES

1. Reed SG, Orr MT, Fox CB. Key roles of adjuvants in modern vaccines. Nat Med. 2013;19(12):1597-608.

2. NIAID. 2018 NIAID Strategic Plan for Research on Vaccine Adjuvants [Available from: https://www.niaid.nih.gov/sites/default/files/NIAIDStrategicPlanVaccineAdjuvants2018.pdf.

3. Pasquale AD, Preiss S, Silva FTD, Garçon N. Vaccine adjuvants: from 1920 to 2015 and beyond. Vaccines. 2015;3(2):320-43.

4. Schmader K. In the Clinic-Herpes Zoster (vol 169, pg ITC19, 2018). ANNALS OF INTERNAL MEDICINE. 2018;169(7):516-.

5. Harpaz R, Ortega-Sanchez IR, Seward JF. Prevention of herpes zoster: recommendations of the Advisory Committee on Immunization Practices (ACIP). Morbidity and Mortality Weekly Report: Recommendations and Reports. 2008;57(5):1-30.

6. Dooling KL, Guo A, Patel M, Lee GM, Moore K, Belongia EA, et al. Recommendations of the Advisory Committee on Immunization Practices for use of herpes zoster vaccines. Morbidity and Mortality Weekly Report. 2018;67(3):103.

7. Shingrix Package Insert [Available from: https://www.fda.gov/media/108597/download.

8. Cunningham AL, Lal H, Kovac M, Chlibek R, Hwang S-J, Díez-Domingo J, et al. Efficacy of the herpes zoster subunit vaccine in adults 70 years of age or older. New England Journal of Medicine. 2016;375(11):1019-32.

9. Lal H, Cunningham AL, Godeaux O, Chlibek R, Diez-Domingo J, Hwang S-J, et al. Efficacy of an adjuvanted herpes zoster subunit vaccine in older adults. New England Journal of Medicine. 2015;372(22):2087-96.

10. Deng Y, Jing Y, Campbell AE, Gravenstein S. Age-related impaired type 1 T cell responses to influenza: reduced activation ex vivo, decreased expansion in CTL culture in vitro, and blunted response to influenza vaccination in vivo in the elderly. The Journal of Immunology. 2004;172(6):3437-46.

11. Smeeth L, Thomas SL, Hall AJ, Hubbard R, Farrington P, Vallance P. Risk of myocardial infarction and stroke after acute infection or vaccination. New England Journal of Medicine. 2004;351(25):2611-8.

12. Shahid Z, Kleppinger A, Gentleman B, Falsey AR, McElhaney JE. Clinical and immunologic predictors of influenza illness among vaccinated older adults. Vaccine. 2010;28(38):6145-51.

13. Grohskopf LA, Alyanak E, Broder KR, Walter EB, Fry AM, Jernigan DB. Prevention and control of seasonal influenza with vaccines: recommendations of the Advisory Committee on Immunization Practices—United States, 2019–20 influenza season. MMWR Recommendations and reports. 2019;68(3):1.

14. Fluzone High-Dose Quadrivalent Package Insert [Available from: https://www.fda.gov/media/132238/download.

15. Fluzone High-Dose Package Insert [Available from: https://www.fda.gov/media/119870/download.

16. FLUAD Package Insert [Available from: https://www.fda.gov/media/94583/download.

17. Falsey AR, Treanor JJ, Tornieporth N, Capellan J, Gorse GJ. Randomized, double-blind controlled phase 3 trial comparing the immunogenicity of high-dose and standard-dose influenza vaccine in adults 65 years of age and older. The Journal of infectious diseases. 2009;200(2):172-80.

18. Liu C, Schmader K, Harrington T, Roundtree W, Auerbach H, Walter E, et al., editors. Comparative Safety of Adjuvanted versus High-Dose Inactivated Influenza Vaccines in Older Adults. JOURNAL OF THE AMERICAN GERIATRICS SOCIETY; 2020: WILEY 111 RIVER ST, HOBOKEN 07030-5774, NJ USA.

19. ACIP. ACIP Summary Report, February 27-28, 2019 [Available from: https://www.cdc.gov/vaccines/acip/meetings/downloads/min-archive/min-2019-02-508.pdf.

20. Izurieta HS, Chillarige Y, Kelman J, Wei Y, Lu Y, Xu W, et al. Relative effectiveness of cell-cultured and egg-based influenza vaccines among elderly persons in the United States, 2017–2018. The Journal of infectious diseases. 2019;220(8):1255-64.

21. Kroger AT, Duchin JS, Vázquez M. General best practice guidelines for immunization: best practices guidance of the Advisory Committee on Immunization Practices (ACIP). 1917.

22. Schwarz TF, Aggarwal N, Moeckesch B, Schenkenberger I, Claeys C, Douha M, et al. Immunogenicity and safety of an adjuvanted herpes zoster subunit vaccine coadministered with seasonal influenza vaccine in adults aged 50 years or older. The Journal of infectious diseases. 2017;216(11):1352-61.

23. Brazier J, Jones N, Kind P. Testing the validity of the Euroqol and comparing it with the SF-36 health survey questionnaire. Qual Life Res. 1993;2(3):169-80.

24. Herdman M, Gudex C, Lloyd A, Janssen M, Kind P, Parkin D, et al. Development and preliminary testing of the new five-level version of EQ-5D (EQ-5D-5L). Qual Life Res. 2011;20(10):1727-36.

25. van Hout B, Janssen MF, Feng YS, Kohlmann T, Busschbach J, Golicki D, et al. Interim scoring for the EQ-5D-5L: mapping the EQ-5D-5L to EQ-5D-3L value sets. Value Health. 2012;15(5):708-15.

26. Gandek B, Ware JE, Aaronson NK, Apolone G, Bjorner JB, Brazier JE, et al. Cross-validation of item selection and scoring for the SF-12 Health Survey in nine countries: results from the IQOLA Project. International Quality of Life Assessment. J Clin Epidemiol. 1998;51(11):1171-8.

27. Ware J, Jr., Kosinski M, Keller SD. A 12-Item Short-Form Health Survey: construction of scales and preliminary tests of reliability and validity. Med Care. 1996;34(3):220-33.

28. Katz JM, Hancock K, Xu X. Serologic assays for influenza surveillance, diagnosis and vaccine evaluation. Expert Rev Anti Infect Ther. 2011;9(6):669-83.

29. Yan X, Su XG. Stratified Wilson and Newcombe Confidence Intervals for Multiple Binomial Proportions. Stat Biopharm Res. 2010;2(3):329-35.

30. Grohskopf LA, Sokolow LZ, Broder KR, Walter EB, Bresee JS, Fry AM, et al. Prevention and Control of Seasonal Influenza with Vaccines: Recommendations of the Advisory Committee on Immunization Practices - United States, 2017-18 Influenza Season. MMWR Recomm Rep. 2017;66(2):1-20.

31. Kwong JC, Vasa PP, Campitelli MA, Hawken S, Wilson K, Rosella LC, et al. Risk of Guillain-Barre syndrome after seasonal influenza vaccination and influenza health-care encounters: a self-controlled study. Lancet Infect Dis. 2013;13(9):769-76.

32. McNeil MM, Weintraub ES, Duffy J, Sukumaran L, Jacobsen SJ, Klein NP, et al. Risk of anaphylaxis after vaccination in children and adults. J Allergy Clin Immunol. 2016;137(3):868-78.

33. ACIP. Risk of Guillain-Barre Syndrome (GBS) after Recombinant Zoster Vaccine (RZV), February 24-25, 2021 [updated Available from: https://www.cdc.gov/vaccines/acip/meetings/downloads/slides-2021-02/24-25/02-Zoster-Vaccines-Forshee.pdf.

Appendix
