# Supplementary material for: Safety of Simultaneous Vaccination With Adjuvanted Zoster Vaccine and Adjuvanted Influenza Vaccine: A Randomized Clinical Trial
Source: JAMA Netw Open. 2024 Oct 24;7(10):e2440817. doi: 10.1001/jamanetworkopen.2024.40817 (PMC11581605; doi:10.1001/jamanetworkopen.2024.40817)
Supplement: Supplement 2. — eMethods 1. Eligibility Criteria eTable 1. Reactogenicity Assessment eMethods 2. EQ-5D-5L and VAS eTable 2. Local and Systemic Reactions During Days 1 Through 8 Following RZV Dose 1 and aIIV4 vs RZV Dose 1 and HD-IIV4 in Older Adults Aged ≥65 Years eTable 3. Proportion of Participants With at Least One Severe (Grade 3) Solicited Local or Systemic Reactogenicity Event on Days 1-8 After RZV Dose 2 in the RZV and aIIV4 Group vs RZV and HD-IIV4 Group eTable 4. Proportion of Participants With Moderate-to-Severe (Grade 2-3) Solicited Local or Systemic Reactogenicity Events on Days 1-8 After RZV Dose 1 and After RZV Dose 2 in the RZV and aIIV4 Group vs RZV and HD-IIV4 Group eTable 5. Proportion of Participants With at Least One Severe (Grade 3) Solicited Local or Systemic Reactogenicity Event on Days 1-8 After RZV Dose 1 in Each Study Group by Age, 65-69 or ≥70 Years eFigure 1. Changes in Health-Related Quality of Life as Measured by EQ-5D-5L Index After RZV Dose 1 and aIIV4 With RZV Dose 1 and HD-IIV4 eFigure 2. Changes in Health-Related Quality of Life as Measured by EQ Visual Analogue Scale (VAS) After RZV Dose 1 and aIIV4 With RZV Dose 1 and HD-IIV4 [file jamanetwopen-e2440817-s002.pdf]

Supplementary Online Content

Schmader KE, Walter EB, Talaat KR, et al. Safety of simultaneous vaccination with adjuvanted zoster vaccine and adjuvanted influenza vaccine: a randomized clinical trial. *JAMA Netw Open.* 2024;7(10):e2440817. doi:10.1001/jamanetworkopen.2024.40817

eMethods 1. Eligibility Criteria

eTable 1. Reactogenicity Assessment

eMethods 2. EQ-5D-5L and VAS

eTable 2. Local and Systemic Reactions During Days 1 Through 8 Following RZV Dose 1 and aIIV4 vs RZV Dose 1 and HD-IIV4 in Older Adults Aged ≥ 65 Years

eTable 3. Proportion of Participants With at Least One Severe (Grade 3) Solicited Local or Systemic Reactogenicity Event on Days 1-8 After RZV Dose 2 in the RZV and aIIV4 Group vs RZV and HD-IIV4 Group

eTable 4. Proportion of Participants With Moderate-to-Severe (Grade 2-3) Solicited Local or Systemic Reactogenicity Events on Days 1-8 After RZV Dose 1 and After RZV Dose 2 in the RZV and aIIV4 Group vs RZV and HD-IIV4 Group

eTable 5. Proportion of Participants With at Least One Severe (Grade 3) Solicited Local or Systemic Reactogenicity Event on Days 1-8 After RZV Dose 1 in Each Study Group by Age, 65-69 or ≥70 Years

eFigure 1. Changes in Health-Related Quality of Life as Measured by EQ-5D-5L Index After RZV Dose 1 and aIIV4 With RZV Dose 1 and HD-IIV4

eFigure 2. Changes in Health-Related Quality of Life as Measured by EQ Visual Analogue Scale (VAS) After RZV Dose 1 and aIIV4 With RZV Dose 1 and HD-IIV4

This supplementary material has been provided by the authors to give readers additional information about their work.

© 2024 Schmader KE et al. *JAMA Network Open.*

eMethods 1. Eligibility Criteria

Subject Inclusion Criteria

- Male or female age ≥ 65 years
- Intention of receiving IIV and RZV based on ACIP-CDC guidelines
- Able to speak English
- Willing to provide written informed consent
- Living in the community
- Intention of being available for entire study period and complete all relevant study procedures, including follow-up phone calls and clinic visits.
- If HIV positive, HIV should be clinically stable

Subject Exclusion Criteria

- IIV or recombinant influenza vaccine (RIV) receipt during the respective 2021-2022 or 2022-2023 influenza season prior to study enrollment
- Prior receipt of recombinant zoster vaccine (Shingrix)
- For non-COVID-19 Vaccines:
  - Receipt of any inactivated vaccine within 2 weeks prior to enrollment in this study
  - Receipt of any live vaccine within 4 weeks prior to enrollment in this study
  - Planning receipt of any non-COVID-19 vaccine during the entire period
- For COVID-19 Vaccines:
  - Receipt of COVID-19 vaccine within 2 weeks prior to enrollment in this study. For those who have initiated a COVID-19 vaccine series, enrollment is not allowed until 2 weeks after the final dose of a COVID-19 vaccine is completed.
  - Planning receipt of a COVID-19 vaccine within 2 weeks after administration of study influenza and first dose recombinant zoster study vaccines.
- Have acute illness or exacerbation of chronic illness within 72 hours of study vaccination
- Hospitalization within the last 30 days for any reason
- History of febrile illness (> 100.0°F or 37.8°C) within the past 24 hours prior to IIV administration
- Has immunosuppression as a result of an underlying illness or treatment, or use of chemotherapy or radiation therapy within the preceding 12 months
- Has an active neoplastic disease (excluding non-melanoma skin cancer or prostate cancer that is stable in the absence of therapy) *Participants with a history of malignancy may be included if, after previous treatment by surgical excision, chemotherapy or radiation therapy, the participant has been observed for a period that in the investigator's estimation provides a reasonable assurance of sustained cure
- A history of autoimmune disease, that requires immunosuppressive agents or any other chronic medical condition considered clinically significant by the investigator
- Use of chronic oral or intravenous administration (≥14 days) of immunosuppressive doses of steroids, i.e., prednisone >10 mg per day, immunosuppressants or other immune-modifying drugs within 30 days of starting this study. (Use of topical, nasal, or inhaled steroids is permitted)
- Thrombocytopenia, bleeding disorder, or anticoagulant use contraindicating intramuscular injection (a daily aspirin may be acceptable)
- Contraindication to IIV receipt including history of severe allergic reaction after a previous dose of any influenza vaccine; or to a vaccine component, including egg protein
- Contraindication to RZV including history of a severe allergic reaction to any component of the RZV vaccine (including saponin or polysorbate 80) or to dose 2 of RZV
- History of Guillain-Barré syndrome
- History of Hepatitis C or active Hepatitis B

© 2024 Schmader KE et al. *JAMA Network Open.*

- Receipt of blood or blood-derived products (including immunoglobulin) within 6 months prior to study vaccination
- Dementia, any cognitive condition, or substance abuse that could interfere with study compliance
- Anyone who is already enrolled or plans to enroll in another clinical trial with an investigational product within 28 days of vaccine receipt. Co-enrollment in observational or behavioral intervention studies are allowed at any time while enrollment in a clinical trial involving an investigational product (other than vaccine) may occur after 28 days following vaccine receipt
- Any condition which, in the opinion of the investigators, may pose a health risk to the subject or interfere with the evaluation of the study objectives
- Anyone who is a relative of any research study personnel
- Anyone who is an employee of any research study personnel

© 2024 Schmader KE et al. *JAMA Network Open.*

eTable 1. Reactogenicity Assessment

The occurrence of solicited reactogenicity events and unsolicited adverse events was assessed daily through post-vaccination Day 8 using a standard symptom diary. The severity grading is described in the Tables below.

Solicited Injection-site Reactogenicity

Symptom	Mild (Grade 1)	Moderate (Grade 2)	Severe (Grade 3)
Pain	Any pain neither interfering with nor preventing normal every day activities.	Painful when limb is moved and interferes with every day activities.	Significant pain at rest. Prevents normal every day activities.
Induration/ Swelling	≥ 20 mm to ≤ 50 mm diameter	> 50 mm to ≤ 100 mm diameter	> 100 mm diameter
Erythema (Redness)	≥ 20 mm to ≤ 50 mm diameter	> 50 mm to ≤ 100 mm diameter	> 100 mm diameter

Solicited Systemic Reactogenicity

Systemic	Mild (Grade 1)	Moderate (Grade 2)	Severe (Grade 3)
Fever (°C)	≥ 37.5 - < 38.4° C<br>≥ 100.0 - < 101.1° F	≥ 38.4 - < 39° C<br>≥ 101.1 - < 102.2° F	≥ 39° C<br>≥ 102.2° F
Fatigue/ Malaise	Fatigue that is easily tolerated	Fatigue that interferes with normal activity	Fatigue that prevents normal activity
Myalgia	Myalgia that is easily tolerated	Myalgia that interferes with normal activity	Myalgia that prevents normal activity
Arthralgia	Arthralgia that is easily tolerated	Arthralgia that interferes with normal activity	Arthralgia that prevents normal activity
Headache	Headache that is easily tolerated	Headache that interferes with normal activity	Headache that prevents normal activity
Gastrointestinal symptoms (nausea, vomiting, diarrhea, and/or abdominal pain)	Gastrointestinal symptoms that are easily tolerated	Gastrointestinal symptoms that interfere with normal activity	Gastrointestinal symptoms that prevent normal activity
Chills/Shivering	Shivering that is easily tolerated	Shivering that interferes with normal activity	Shivering that prevents normal activity

© 2024 Schmader KE et al. *JAMA Network Open.*

eMethods 2. EQ-5D-5L and VAS

Under each heading, please check the ONE box that best describes your health TODAY.

MOBILITY

I have no problems walking ❑

I have slight problems walking ❑

I have moderate problems walking ❑

I have severe problems walking ❑

I am unable to walk ❑

SELF-CARE

I have no problems washing or dressing myself ❑

I have slight problems washing or dressing myself ❑

I have moderate problems washing or dressing myself ❑

I have severe problems washing or dressing myself ❑

I am unable to wash or dress myself ❑

USUAL ACTIVITIES *(e.g. work, study, housework, family or leisure activities)*

I have no problems doing my usual activities ❑

I have slight problems doing my usual activities ❑

I have moderate problems doing my usual activities ❑

I have severe problems doing my usual activities ❑

I am unable to do my usual activities ❑

PAIN / DISCOMFORT

I have no pain or discomfort ❑

I have slight pain or discomfort ❑

I have moderate pain or discomfort ❑

I have severe pain or discomfort ❑

I have extreme pain or discomfort ❑

ANXIETY / DEPRESSION

I am not anxious or depressed ❑

I am slightly anxious or depressed ❑

I am moderately anxious or depressed ❑

I am severely anxious or depressed ❑

I am extremely anxious or depressed ❑

© 2024 Schmader KE et al. *JAMA Network Open.*

EQ- VAS

- We would like to know how good or bad your health is TODAY.
- This scale is numbered from 0 to 100.
- 100 means the best health you can imagine.
  0 means the worst health you can imagine.
- Mark an X on the scale to indicate how your health is TODAY.
- Now, please write the number you marked on the scale in the box below.

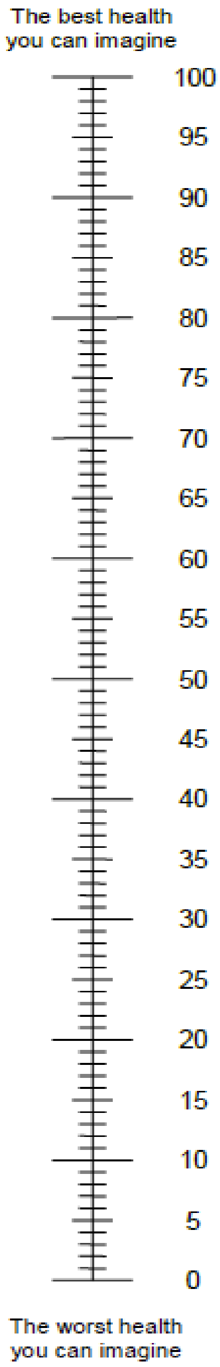

YOUR HEALTH TODAY = [ ]

© 2024 Schmader KE et al. *JAMA Network Open.*

eTable 2. Local and Systemic Reactions During Days 1 Through 8 Following RZV Dose 1 and aIIV4 vs RZV Dose 1 and HD-IIV4 in Older Adults Aged ≥ 65 Years

Reaction	Group	None		Mild		Moderate		Severe		Moderate/Severe	
		N	%	N	%	N	%	N	%	N	%
Local											
Pain: RZV Vaccine	aIIV4	25	19.2	67	51.5	33	25.4	5	3.8	38	29.2
	HD-IIV4	23	16.9	73	53.7	36	26.5	4	2.9	40	29.4
Pain: Flu Vaccine	aIIV4	60	46.2	58	44.6	10	7.7	2	1.5	12	9.2
	HD-IIV4	46	33.8	70	51.5	19	14.0	1	0.7	20	14.7
Swelling: RZV Vaccine	aIIV4	100	76.9	17	13.1	12	9.2	1	0.8	13	10.0
	HD-IIV4	112	82.4	14	10.3	10	7.4	0	0.0	10	7.4
Swelling: Flu Vaccine	aIIV4	115	88.5	12	9.2	3	2.3	0	0.0	3	2.3
	HD-IIV4	119	87.5	10	7.4	6	4.4	1	0.7	7	5.1
Redness: RZV Vaccine	aIIV4	94	72.3	17	13.1	16	12.3	3	2.3	19	14.6
	HD-IIV4	98	72.1	13	9.6	24	17.6	1	0.7	25	18.4
Redness: Flu Vaccine	aIIV4	118	90.8	8	6.2	4	3.1	0	0.0	4	3.1
	HD-IIV4	119	87.5	12	8.8	4	2.9	1	0.7	5	3.7
Systemic											
Fatigue	aIIV4	75	57.7	26	20.0	25	19.2	4	3.1	29	22.3
	HD-IIV4	66	48.5	37	27.2	27	19.9	6	4.4	33	24.3
Myalgia	aIIV4	76	58.5	31	23.8	22	16.9	1	0.8	23	17.7
	HD-IIV4	71	52.2	40	29.4	20	14.7	5	3.7	25	18.4
Headache	aIIV4	85	65.4	33	25.4	10	7.7	2	1.5	12	9.2
	HD-IIV4	87	64.0	36	26.5	13	9.6	0	0.0	13	9.6
Arthralgia	aIIV4	102	78.5	22	16.9	5	3.8	1	0.8	6	4.6
	HD-IIV4	105	77.2	18	13.2	9	6.6	4	2.9	13	9.6
Fever	aIIV4	120	92.3	8	6.2	1	0.8	1	0.8	2	1.5
	HD-IIV4	125	92.6	10	7.4	0	0.0	0	0.0	0	0.0
Chills	aIIV4	106	81.5	16	12.3	7	5.4	1	0.8	8	6.2
	HD-IIV4	105	77.2	19	14.0	10	7.4	2	1.5	12	8.8
Nausea	aIIV4	121	93.1	7	5.4	2	1.5	0	0.0	2	1.5
	HD-IIV4	127	93.4	4	2.9	4	2.9	1	0.7	5	3.7
Vomiting	aIIV4	130	100.0	0	0.0	0	0.0	0	0.0	0	0.0
	HD-IIV4	135	99.3	0	0.0	0	0.0	1	0.7	1	0.7
Diarrhea	aIIV4	122	93.8	5	3.8	2	1.5	1	0.8	3	2.3
	HD-IIV4	131	96.3	4	2.9	1	0.7	0	0.0	1	0.7
Abdominal Pain	aIIV4	127	97.7	3	2.3	0	0.0	0	0.0	0	0.0
	HD-IIV4	131	96.3	3	2.2	1	0.7	1	0.7	2	1.5

© 2024 Schmader KE et al. *JAMA Network Open.*

eTable 3. Proportion of Participants With at Least One Severe (Grade 3) Solicited Local or Systemic Reactogenicity Event on Days 1-8 After RZV Dose 2 in the RZV and aIIV4 Group vs RZV and HD-IIV4 Group

Reaction	Group	No		Yes		Noninferiority Test 10% Margin			
All Reactogenicity Events		N	%	N	%	Diff	Lower CI	Upper CI	p-value
	aIIV4	118	91.5	11	8.5	.	.	.	.
	HD-IIV4	114	85.1	20	14.9	-6.3%	-14.4	1.6	<0.0001
Local									
	aIIV4	123	95.4	6	4.7	.	.	.	.
	HD-IIV4	126	94.0	8	6.0	-1.2%	-7.6	4.6	0.0001
Systemic									
	aIIV4	120	93.0	9	7.0				
	HD-IIV4	120	89.6	14	10.5	-3.4%	-10.8	3.6	0.0001

© 2024 Schmader KE et al. *JAMA Network Open.*

eTable 4. Proportion of Participants With Moderate-to-Severe (Grade 2-3) Solicited Local or Systemic Reactogenicity Events on Days 1-8 After RZV Dose 1 and After RZV Dose 2 in the RZV and aIIV4 Group vs RZV and HD-IIV4 Group

Reaction	Group	No		Yes		Noninferiority Test 10% Margin			
Grade 2-3 Reactogenicity Events after RZV Dose 1		N	%	N	%	Diff	Lower CI	Upper CI	p-value
	aIIV4	57	43.9	73	56.2				
	HD-IIV4	67	49.3	69	50.7	5.4%	-6.5	17.1	0.2235
Grade 2-3 Reactogenicity Events after RZV Dose 2									
	aIIV4	58	45.0	71	55.0				
	HD-IIV4	61	45.5	73	54.5	0.7%	-11.1	12.6	0.0625

© 2024 Schmader KE et al. *JAMA Network Open.*

eTable 5. Proportion of Participants With at Least One Severe (Grade 3) Solicited Local or Systemic Reactogenicity Event on Days 1-8 After RZV Dose 1 in Each Study Group by Age, 65-69 or ≥70 Years

	Age 65-69 Years		aIIV4 minus HD-IIV4 95% CI of the Difference
	No	Yes	
Group	N (%)	N (%)	
aIIV4	41 (93.2)	3 (6.8)	
HD-IIV4	41 (85.2)	7 (14.6)	-7.77 (-20.22, 4.69)
	Age 70≥ Years		
	No	Yes	
Group	N (%)	N (%)	
aIIV4	74 (86.1)	12 (13.9)	
HD-IIV4	78 (88.6)	10 (11.3)	2.59 (-7.29, 12.47)

© 2024 Schmader KE et al. *JAMA Network Open.*

eFigure 1. Changes in Health-Related Quality of Life as Measured by EQ-5D-5L Index After RZV Dose 1 and aIIV4 With RZV Dose 1 and HD-IIV4

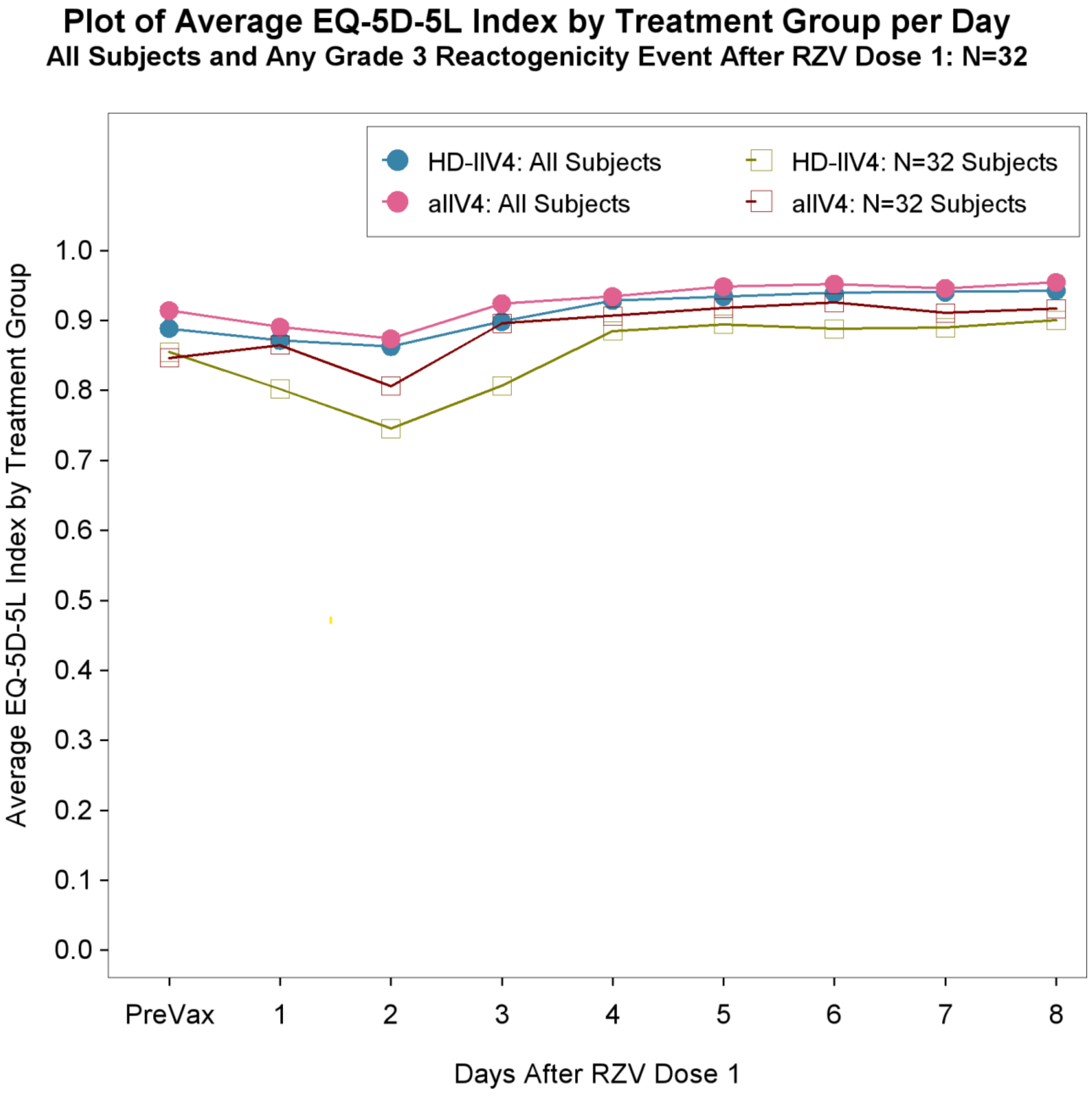

© 2024 Schmader KE et al. *JAMA Network Open.*

eFigure 2. Changes in Health-Related Quality of Life as Measured by EQ Visual Analogue Scale (VAS) After RZV Dose 1 and aIIV4 With RZV Dose 1 and HD-IIV4

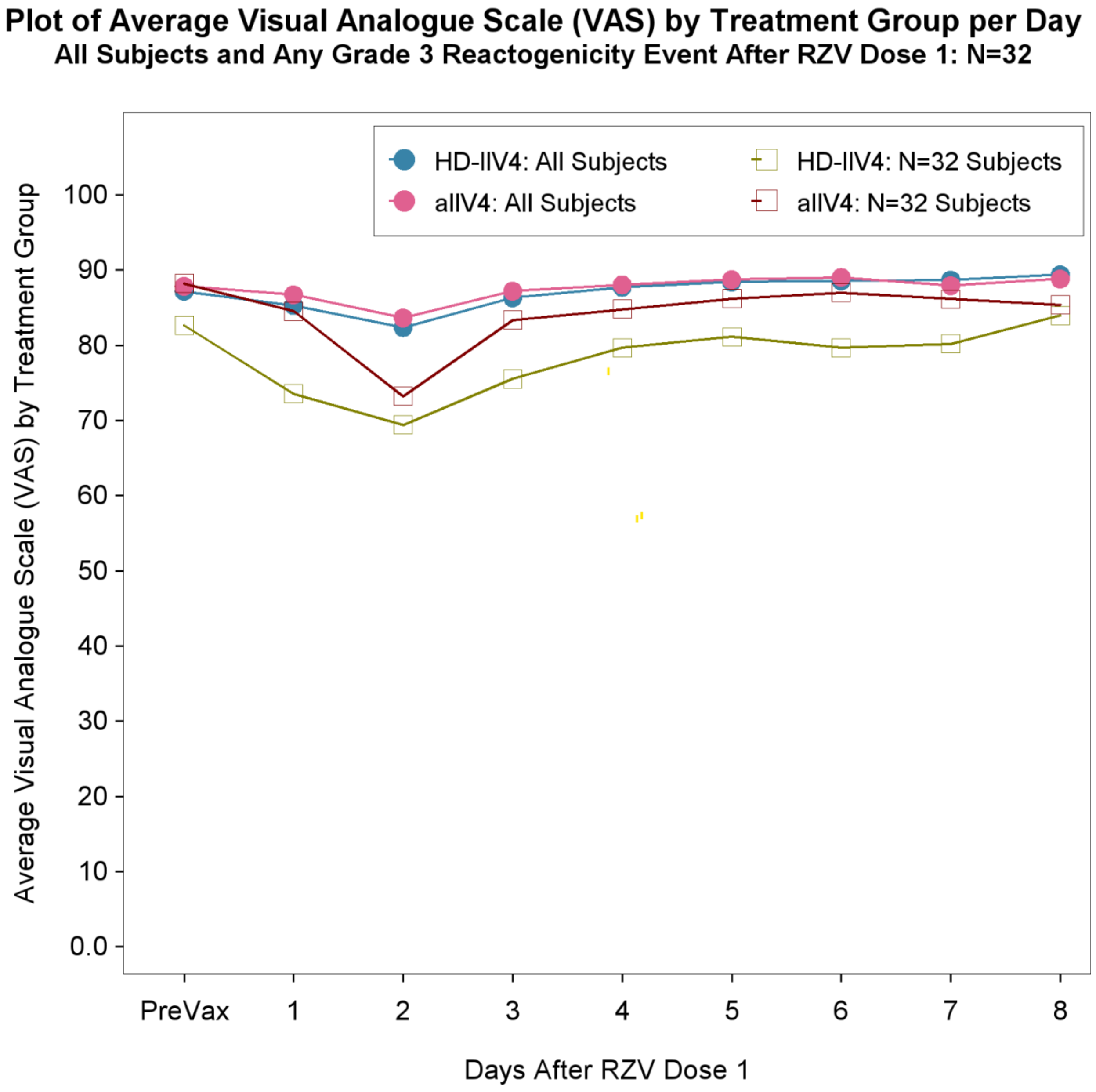

© 2024 Schmader KE et al. *JAMA Network Open.*
