# Supplementary material for: Safety of Simultaneous Vaccination With Adjuvanted Zoster Vaccine and Adjuvanted Influenza Vaccine: A Randomized Clinical Trial
Source: JAMA Netw Open. 2024 Oct 24;7(10):e2440817. doi: 10.1001/jamanetworkopen.2024.40817 (PMC11581605; doi:10.1001/jamanetworkopen.2024.40817)
Supplement: Supplement 3. — Data Sharing Statement [file jamanetwopen-e2440817-s003.pdf]

Data Sharing Statement

Schmader. Safety of Simultaneous Vaccination With Adjuvanted Zoster Vaccine and Adjuvanted Influenza Vaccine. *JAMA Netw Open*. Published October 24, 2024. doi:10.1001/jamanetworkopen.2024.40817

Data
Additional Information: ClinicalTrials.gov Identifier: NCT05007041
Data available: No

Additional Information
Explanation for why data not available: Individual deidentified participant data will not be shared because it was not part of the consent process and the effort required to do this task will not be supported by the sponsor
